# Auditory Brainstem Deficits from Early Treatment with a CSF1R Inhibitor Largely Recover with Microglial Repopulation

**DOI:** 10.1523/ENEURO.0318-20.2021

**Published:** 2021-03-19

**Authors:** Giedre Milinkeviciute, Sima M. Chokr, Karina S. Cramer

**Affiliations:** Department of Neurobiology and Behavior, University of California, Irvine, CA 92697

**Keywords:** auditory brainstem, calyx of Held, depletion, GFAP, microglia, MNTB

## Abstract

Signaling between neurons and glia is necessary for the formation of functional neural circuits. A role for microglia in the maturation of connections in the medial nucleus of the trapezoid body (MNTB) was previously demonstrated by postnatal microglial elimination using a colony stimulating factor 1 receptor (CSF1R). Defective pruning of calyces of Held and significant reduction of the mature astrocyte marker glial fibrillary acidic protein (GFAP) were observed after hearing onset. Here, we investigated the time course required for microglia to populate the mouse MNTB after cessation of CSF1R inhibitor treatment. We then examined whether defects seen after microglial depletion were rectified by microglial repopulation. We found that microglia returned to control levels at four weeks of age (18 d postcessation of treatment). Calyceal innervation of MNTB neurons was comparable to control levels at four weeks and GFAP expression recovered by seven weeks. We further investigated the effects of microglia elimination and repopulation on auditory function using auditory brainstem recordings (ABRs). Temporary microglial depletion significantly elevated auditory thresholds in response to 4. 8, and 12 kHz at four weeks. Treatment significantly affected latencies, interpeak latencies, and amplitudes of all the ABR peaks in response to many of the frequencies tested. These effects largely recovered by seven weeks. These findings highlight the functions of microglia in the formation of auditory neural circuits early in development. Further, the results suggest that microglia retain their developmental functions beyond the period of circuit refinement.

## Significance Statement

Auditory brainstem pathways are optimized for their special functions that are shaped during development, which relies on the functions of non-neuronal cells, such as microglia and astrocytes. When microglia were pharmacologically eliminated during the early postnatal period with a colony stimulating factor 1 receptor (CSF1R) inhibitor, excess calyces were not pruned and astrocytes did not mature properly in the auditory brainstem. Here, we show that once this drug is withdrawn, microglia gradually return to the auditory nuclei. After microglia reemerge in the medial nucleus of the trapezoid body (MNTB), synaptic pruning of calyces of Held resumes and maturation of astrocytes and auditory function recover. The findings suggest that the auditory brainstem pathways can be shaped by microglia even after their normal period of circuit development.

## Introduction

Specialized neural circuits in the auditory brainstem carry out computations needed for auditory processing, including sound source localization (Grothe et al., 2010). Auditory nerve fibers synapse onto bushy cells in the anteroventral cochlear nucleus (AVCN; Held, 1893; Brawer and Morest, 1975; Ryugo and Sento, 1991; Lauer et al., 2013). Globular bushy cell (GBC) axon terminals form the elaborate calyx of Held (Held, 1893), which contacts principal neurons in the contralateral medial nucleus of the trapezoid body (MNTB; Morest, 1968; Smith et al., 1991; Kandler and Friauf, 1993). MNTB neurons inhibit neurons in the lateral superior olive (LSO; Kuwabara and Zook, 1991; Tollin, 2003). LSO neurons also receive tonotopically matched excitatory input from the ipsilateral side and use the balance between inhibition and excitation to compute interaural intensity differences (Boudreau and Tsuchitani, 1968; Sanes and Rubel, 1988; Grothe, 2003). This pathway, which allows for fast and reliable transmission of sound signals (Borst and Sakmann, 1996; Trussell, 1999; Taschenberger et al., 2002), relies on precision in developmental mechanisms.

Microglia mediate major functions in neural development (Reemst et al., 2016; Nelson and Lenz, 2017) and formation of neuronal circuits (Miyamoto et al., 2016; Basilico et al., 2019) through their roles in synapse elimination (Schafer and Stevens, 2010; Paolicelli et al., 2011; Schafer et al., 2012; Neniskyte and Gross, 2017), construction of new synapses (Parkhurst et al., 2013; Miyamoto et al., 2016), and synaptic remodeling (Tremblay et al., 2010; Schafer et al., 2012; Weinhard et al., 2018). When microglial function is disturbed, excess synapses persist in adulthood, resulting in anatomic abnormalities in neural circuits (Kettenmann et al., 2013).

Microglia are sparse in MNTB during the first postnatal week, then peak in density just after hearing onset at two weeks (Dinh et al., 2014). Microglia are found in close proximity to the developing calyx of Held (Holcomb et al., 2013; Dinh et al., 2014). From multiple small “protocalyces” contacting a single MNTB neuron (Kandler and Friauf, 1993), a single dominant calyx of Held emerges and monoinnervation of MNTB neurons is established (Morest, 1968; Sätzler et al., 2002; Hoffpauir et al., 2006; Holcomb et al., 2013). Microglial elimination before hearing onset results in excess polyinnervated neurons, signifying a microglial role in the development of neural circuits in the brainstem (Milinkeviciute et al., 2019).

Like microglia, astrocytes support synaptic development and remodeling. Despite differences between microglia and astrocytes in terms of their origin and structure, these cell types exhibit coordinated responses to insult (Schiweck et al., 2018; Vainchtein and Molofsky, 2020). Astrocytes and microglia communicate through bidirectional signaling and shared responses to environmental signals (Jha et al., 2019; Vainchtein and Molofsky, 2020). Microglia can influence expression of astrocytic proteins (Elmore et al., 2014; Spangenberg et al., 2016; Jin et al., 2017) and astrocytes can signal to microglia through several pathways (Bialas and Stevens, 2013; Vainchtein et al., 2018). In the auditory brainstem, microglial depletion prevented the emergence of astrocytic expression of glial fibrillary acidic protein (GFAP), a marker for mature astrocytes (Wofchuk and Rodnight, 1995; Gomes et al., 1999; Middeldorp and Hol, 2011), further highlighting the relationship between the two glial populations (Milinkeviciute et al., 2019).

Here, we tested whether microglia can return after postnatal pharmacological elimination, and whether microglial repopulation can restore development of auditory brainstem pathways. We further tested whether temporary postnatal microglial elimination has an effect on auditory function. We depleted microglia during the early postnatal period with an inhibitor of colony stimulating factor 1 receptor (CSF1R; Milinkeviciute et al., 2019), which is essential for microglial survival and proliferation (Stanley et al., 1997; Erblich et al., 2011). We then allowed microglia to repopulate by cessation of treatment after postnatal day (P)10. We found that microglia slowly recolonize the brainstem, first in lateral regions and later in progressively more medial regions. We found that defects present at P13 (Milinkeviciute et al., 2019), synaptic pruning and decreased expression of GFAP, were both corrected when microglia repopulated the MNTB. Additionally, we evaluated the overall auditory brainstem function of these animals using ABRs. We found elevated auditory thresholds, increased latencies and interpeak latencies, and decreased amplitudes in almost all ABR peaks in response to most of the frequencies tested at four weeks of age. ABRs were partially restored to normal by seven weeks of age. These results suggest that microglia have early developmental roles in synaptic pruning of calyces of Held, promoting astrocyte maturation, and maturation of auditory brainstem function. Our findings further suggest that microglia can act at later developmental times, at least in part, to restore normal formation of auditory brainstem circuits.

## Materials and Methods

### Animals and treatment

**Figure 1.**
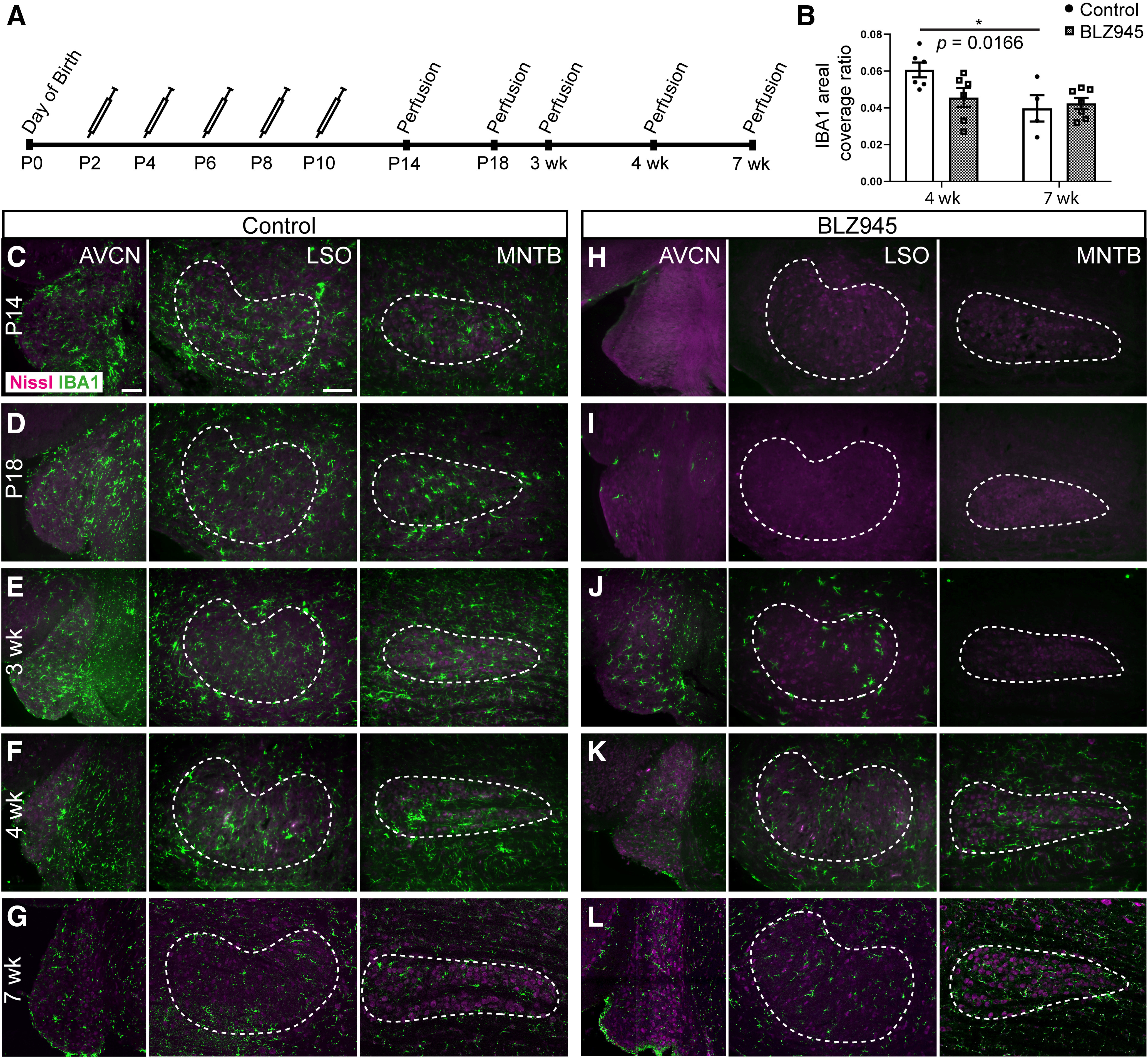
Effects of BLZ945 treatment withdrawal on IBA1 expression in the auditory brainstem. ***A***, The timeline of BLZ945 treatment. ***B***, Areal coverage ratio of IBA1 labeling in MNTB. There was no difference between IBA1 area coverage ratio between control and BLZ945-treated mice at four weeks. With age, IBA1 coverage significantly decreased in control mice only. Row ***C***, Images of IBA1 immunolabel (green) in AVCN, LSO, and MNTB in control mice at P14. LSO and MNTB are indicated by dashed lines. Rows ***D***–***G***, Images of IBA1 immunolabel in AVCN, LSO, and MNTB in control mice at P18, three, four, and seven weeks. At seven weeks, microglia looked sparser in all three brainstem nuclei. Row ***H***, Photographs of IBA1 immunolabel in AVCN, LSO, and MNTB in BLZ945-treated mice at P14. No IBA1-positive cells were observed throughout the auditory brainstem. Row ***I***, Images of IBA1 immunolabel in BLZ945-injected mice at P18 showed only few IBA1-positive cells in AVCN but not LSO and MNTB. Row ***J***, Photographs of BLZ945-treated mice at three weeks show AVCN populated by IBA1-positive cells and few of them were observed in LSO. MNTB was still devoid of microglia. Row ***K***, Photographs of IBA1 immunolabel in AVCN, LSO, and MNTB of BLZ945-treated mice at four weeks. Microglia were spread throughout all the three nuclei. Row ***L***, Photographs of IBA1 labeling in BLZ945-injected animals at seven weeks. IBA1 labeling appeared sparser but was still present in AVCN, LSO, and MNTB. Scale bar in AVCN panel in row ***C***: 100 μm, applies to all AVCN panels across rows ***C–L***. Scale bar in LSO panel in row ***C***: 40 μm, applies to all LSO and MNTB panels across rows ***C–L***; *p <* *0.05.

We used eight P14, seven P18, 18 three-week-old, 37 four-week-old, and 26 seven-week-old wild-type C57BL/6 mice of both sexes. All animal procedures were performed in accordance with the University of California, Irvine animal care committee’s regulations. Mice were housed in groups, with a maximum of five adult mice per cage. Mice were reared in a standard dark/light cycle, fed a standard diet and received food and water *ad libitum*. Whenever possible, mice that were used for ABRs were also used for histology with exception of pruning analysis. Each litter was divided into control and experimental mice. Experimental mice were repeatedly injected with BLZ945 (subcutaneous injections at P2, P4, P6, P8, and P10; MW: 398.48, MedChem Express HY-12768/CS-3971), a small molecule inhibitor of CSF1R (Pyonteck et al., 2013), to eliminate microglia as previously described (Milinkeviciute et al., 2019). BLZ945 was dissolved in dimethylsulfoxide (DMSO; D136-1; Fisher Scientific) and 0.01 ml of solution (200 mg/kg) was administered subcutaneously every 2 d starting from P2 with the last injection delivered at P10. Control mice were injected with DMSO following the same protocol. After cessation of treatment, mice were killed when they were 14 or 18 d or three, four, or seven weeks old (Fig. 1*A*).

### Tissue preparation and immunofluorescence

Mice were weighed and perfused transcardially with 0.9% saline followed by 4% paraformaldehyde (PFA) in 0.1 m phosphate buffer, pH 7.3 (PBS) at three, four, or seven weeks (Fig. 1*A*). Brainstems were dissected and postfixed overnight in PFA solution. Brains were then transferred to a 30% sucrose solution in 0.1 m PBS overnight, embedded in OCT mounting medium, and sectioned coronally at 18 μm using a cryostat (CM 1850-3-1; Leica Microsystems). Tissue was mounted on chrome-alum-coated glass slides in a one-in-five series. Mounted sections were surrounded with a PAP pen hydrophobic barrier and rinsed in 0.1 m PBS for 10 min. Tissue was incubated for 5 min in 0.1% sodium dodecyl sulfate in 0.1 m PBS solution for antigen retrieval followed by 3 10 min washes in 0.1 m PBS. Sections were then blocked with normal goat blocking solution containing 5% normal goat serum (NGS; Vector Laboratories S-1000) and 0.3% Triton X-100 (Acros 9002-93-1) in 0.1 m PBS in a humidity chamber at room temperature. After 1 h, primary antibodies were applied (Table 1). The next day, the tissue was rinsed in 0.1 m PBS and incubated for 1 h in goat anti-rabbit or anti-chicken secondary antibody tagged with an Alexa (Invitrogen) fluorophore (Table 1). Sections were washed in 0.1 m PBS and incubated in blue or red fluorescent Nissl stain (NeuroTrace 435/455 or 530/615, Life Technologies N21482 or N21479) diluted in 1:200 in 0.3% Triton X-100 in 0.1 m PBS for 1 h. Tissue was then rinsed and coverslipped with Glycergel mounting medium (Dako C0563).

**Table 1:** List of antibodies used in the project

Antigen	Host	RRID	Catalog number	Source	Dilution
Primary antibodies					
GFAP	Chicken	AB_304558	ab4674	Abcam	1:1000
IBA1	Rabbit	AB_839504	019-19741	Wako	1:500
VGluT1/2	Rabbit	AB_2285905	135503	Synaptic Systems	1:200
Secondary antibodies					
Alexa Fluor 488	Chicken	AB_2534096	A11039	Thermo Fisher	1:200
Alexa Fluor 647	Rabbit	AB_2535812	A21244	Thermo Fisher	1:500

### Areal coverage analysis

We acquired 20× magnification images of sections throughout the rostro-caudal extent of the MNTB on both sides of the midline using a Zeiss Axioskop-2 microscope, an Axiocam camera, and Axiovision software. For each subject, a series of 20× multichannel fluorescent photographs spanning the rostro-caudal extent of the MNTB was taken and collected as an image stack in FIJI (Schindelin et al., 2012). The Nissl channel was used to outline the MNTB [a region of interest (ROI)] in every section in FIJI. A stack of ROIs from each animal was used to analyze the areal coverage of IBA1 or GFAP immunolabeling in the corresponding MNTB region as described in Milinkeviciute et al. (2019).

### Neuronal tracing and confocal analysis

We used four control and four BLZ-treated mice and five control and five BLZ945-treated mice at three and four weeks, respectively, for neuronal tracing. After transcardial perfusion with artificial CSF (aCSF; 130 mm NaCl, 3 mm KCl, 1.2 mm KH_2_PO_4_, 20 mm NaHCO_3_, 3 mm HEPES, 10 mm glucose, 2 mm CaCl_2_, and 1.3 mm MgSO_4_ perfused with 95% O_2_ and 5% CO_2_), brains were quickly dissected and placed in a chamber with oxygenated aCSF. We temporarily placed the brain in a Petri dish with aCSF and filled calyces of Held in the MNTB with a rhodamine dextran amine (RDA; MW 3000, Invitrogen) solution (6.35% RDA with 0.4% Triton X-100 in PBS). Pulses of RDA were delivered through a pulled glass micropipette in the ventral acoustic stria (VAS) close to the midline using an Electro Square Porator (ECM830; BTX) at a rate of five pulses per second (pps) at 55 V for 50 ms. As a result, axonal projections from the AVCN terminating in calyces of Held in the MNTB were sparsely labeled with RDA on both sides of the brainstem. The brain was then transferred back into the aCSF chamber for ∼2 h under continuous oxygenation to allow dye transport along the GBC axons. The brain was then placed in 4% PFA overnight then transferred to 30% sucrose in 0.1 m PBS and later cryosectioned in the coronal plane at 18 μm. Sections were mounted on chrome-alum-coated glass slides in a one-in-five series and were immunolabeled with anti-VGLUT1/2 antibody (Table 1) following the protocol described above.

Stained slides were analyzed using confocal microscopy (Leica SP8, 63× oil objective, zoom: 1.5, pinhole: 1). Nissl, RDA, and VGLUT1/2 z-stack images of calyces of Held were acquired at a resolution of 1024 × 1024 pixels, with a z-step size of 0.5 μm (Grande et al., 2014; Wang et al., 2018; Milinkeviciute et al., 2019). Gain and offset were set for each fluorescent channel and each slide separately and adjusted if labeling intensity was noticeably different between sections on the same slide. Calyces throughout the MNTB were sparsely and randomly labeled with RDA, thus, the entire mediolateral extent of the MNTB was randomly sampled. If the number of labeled calyces from one slide was too small (<3), an additional slide was used (Table 2).

**Table 2:** Numbers of calyces of Held analyzed and polyinnervated neurons

Treatment	Animals used	Age (weeks)	Animal ID	Reconstructed calyces	Number of polyinnervated neurons	% of polyinnervated neurons
Control	4	3	B987	9	1	11.11
			B989	8	0	0
			B991	13	0	0
			B994	7	2	28.57
BLZ945	4		B990	9	5	55.56
			B992	9	1	11.11
			B993	13	5	38.46
			B995	10	5	50
Control	5	4	B886	8	0	0
			B887	7	1	14.29
			B888	8	0	0
			B894	9	0	0
			B896	9	1	11.11
BLZ945	5		B889	14	4	28.57
			B890	10	3	30
			B891	13	1	7.69
			B895	14	0	0
			B897	12	2	16.67

Image stacks were analyzed using the surface module in Imaris software (v9.5.1; Bitplane). Surfaces of RDA-filled calyces that were complete or near complete and that had adequate VGLUT1/2 immunofluorescence as well as a visible preterminal axon segment were reconstructed using a 0.4 surface detail and 0.4 background subtraction settings. These settings were determined before the analysis and were chosen so as to most reliably reconstruct the surface of the calyx of Held. If necessary, the rendering was adjusted manually to achieve an accurate RDA fill. Calyceal surface area as well as volume were recorded and used to calculate the shape factor of calyces from monoinnervated neurons. A shape factor was calculated as (SA1.5)/(6$\sqrt{\pi }$V) where SA is the surface area of the calyx and V is its volume (DeHoff, 1978; Muniak et al., 2018). A shape factor of 1.0 characterizes a perfect sphere. A large shape factor corresponds with more complex structure, which, in the case of calyx of Held, represents its intricate branching. The analysis was performed blind to the animal treatment group.

### Determination of monoinnervation and polyinnervation

**Figure 2.**
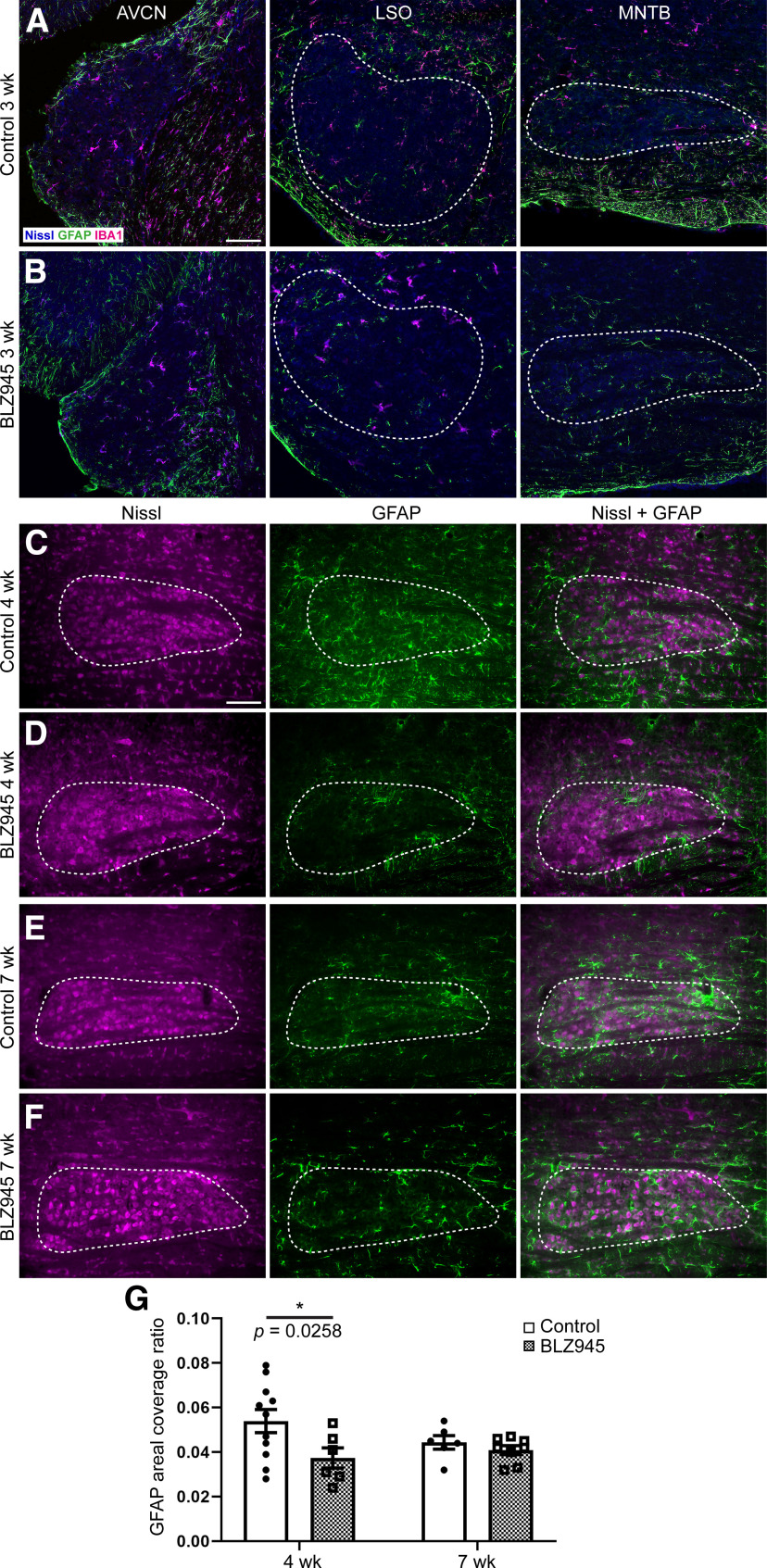
Effects of BLZ945 treatment withdrawal on GFAP expression in the auditory brainstem. Row ***A***, Images of GFAP (green) and IBA1 (magenta) immunolabel in AVCN, LSO, and MNTB in control mice at three weeks. Dashed lines indicate LSO and MNTB. GFAP expression was dense along outer edges of AVCN but sparse within the nucleus. GFAP expression was also sparse in LSO, but denser staining was observed in MNTB. Row ***B***, Images of GFAP and IBA1 immunolabel in AVCN, LSO, and MNTB in BLZ945-treated mice at three weeks. GFAP immunolabel was seen along the outer edges of AVCN and was sparse in LSO. Some GFAP immunoloabel was seen in MNTB. Row ***C***, Images of Nissl, GFAP immunolabel and an overlay of both in MNTB in control mice at four weeks. GFAP expression extended throughout the MNTB. Row ***D***, Images of Nissl, GFAP immunolabel and an overlay of both in MNTB in BLZ945-treated mice at four weeks. GFAP expression was denser than at three weeks but was localized more to the boundaries of MNTB. Row ***E***, ***F***, Images of MNTB in control and BLZ945-injected mice, respectively, at seven weeks. GFAP immunolabeling was pronounced and found throughout MNTB. ***G***, Areal coverage of GFAP expression in MNTB at four and seven weeks. GFAP expression was significantly decreased in BLZ945-treated animals at four weeks compared with age-matched controls. No difference was found between the two experimental groups at seven weeks, and there were no age-related changes in GFAP areal coverage ratio in control or BLZ945-injected mice. Scale bar in row ***A***: 100 μm, applies to all panels in rows ***A***, ***B***. Scale bar in row ***C***: 100 μm, applies to all panels in rows ***C–F***; *p <* *0.05.

The monoinnervation or polyinnervation status of MNTB neurons was evaluated during the reconstruction of RDA-filled calyces of Held. Neurons were classified as monoinnervated if the neuron was contacted only by a single RDA-labeled calyx, with no VGLUT1/2 immunolabel seen outside this calyx. Neurons were classified as polyinnervated if VGLUT1/2 immunolabel was present on the MNTB cell outside of the RDA-filled calyx. To be considered additional calyceal input, the VGLUT1/2 immunolabel outside of the RDA-filled calyx covered at least 25% of the MNTB neuron surface. The threshold of 25% was chosen because the smallest RDA-filled calyx detected across all the 10 analyzed animals contacted ∼25% of the neuronal surface area (Fig. 2). We then determined the percentage of analyzed MNTB neurons that were polyinnervated for the control and BLZ945-treated groups (Table 2).

### Auditory brainstem recordings (ABRs)

ABRs were performed on 13 control and 14 BLZ945-treated mice at four weeks and on 11 control and 15 BLZ945 injected animals at seven weeks. Mice were anesthetized with an intramuscular injection of ketamine (75 mg/kg, KetaVed, VEDCO) and xylazine (15 mg/kg, AnaSed, NADA# 139-236). Body temperature was maintained at 35°C via a far infrared warming pad (Kent Scientific, RT-0501). Sterile ocular lubricant (Puralube Vet Ointment, 006PHM02-1-8) was applied on the eyes following ketamine/xylazine injection. Three pin electrodes were inserted subcutaneously. The positive, negative and ground electrodes were positioned at the vertex, the right cheek, and in the back near the right leg, respectively. Electrodes were connected to a Tucker-Davis Technologies (TDT) RA4PA 4-channel Medusa amplifier, which was connected to a TDT RA16 Medusa Base Station.

ABRs were performed in a sound-attenuating chamber (102 × 98 × 81 cm, Industrial Acoustics Company). Sound stimuli were generated using TDT SigGen software version 4.4 and presented 500 times at a rate of 21 stimuli per second using TDT MF1 Multi-Function Speaker through an ear tube inserted in the animal’s left ear. Acoustic stimuli were emitted via a TDT RP2.1 enhanced real time processor. We controlled the sound level via a TDT PA5 programmable attenuator. Recorded responses were amplified by a TDT SA1 stereo power amp, filtered with the control of BioSig software version 4.4. ABRs were recorded for 12 ms in response to 100-μs click stimuli or 3-ms pure tone stimuli (4, 8, 12, 16, 24, 32 kHz) at decreasing sound levels (5 dB SPL steps from 80 to 10 dB SPL). An averaged response was computed at each sound level and used for analysis.

### ABR analysis

ABRs were analyzed for threshold, peak latency, interpeak latency, and peak amplitude. We computed the mean noise level from the first 20 data points, before the ABR trace. Threshold was defined as the lowest sound level at which the peak I level (μV) was ≥4 SDs above the noise level (Bogaerts et al., 2009). Peaks were manually determined by a blinded observer, and data were recorded to a spreadsheet in BioSig from which the following analyses were performed. Absolute peak latency was determined as the time period from the sound onset to the apex of the peak. Interpeak latency was measured by calculating the difference between absolute peak latencies between peaks I–II, II–III, III–IV, I–III, and I–IV. Peak amplitude was determined as the change in microvolts (μV) between the preceding trough and apex of the peak. Simple linear regressions of input-output functions of absolute peak latencies, interpeak latencies, and trough-to-peak amplitudes at recorded frequency and intensity levels were analyzed. Input-output functions were only analyzed if at least three animals per group were at or above threshold (Tables 3, 4, 5).

**Table 3:** Descriptive statistics of immunostaining and confocal data

Analysis	Mean ± SEM	Treatment
IBA1 (two-way ANOVA, Sidak’s multiple comparisons test)	4 weeks: Control 0.06 ± 0.004 (*n* = 6), BLZ945 0.05 ± 0.01 (*n* = 6); 7 weeks: Control 0.04 ± 0.01 (*n* = 4), BLZ945 0.04 ± 0.003 (*n* = 7)	4-week Control-BLZ945: *p *=* *0.0574, *t *=* *2.36, df = 19; 7-week Control-BLZ945: *p *=* *0.9113, *t *=* *0.388, df = 19; Control 4–7 weeks: *p *=* *0.0166, *t *=* *2.943, df = 19; BLZ945 4–7 weeks: *p *=* *0.8425, *t *=* *0.529, df = 19

GFAP (two-way ANOVA, Sidak’s multiple comparisons test)	4 weeks: Control 0.05 ± 0.005 (*n* = 11, BLZ945 0.04 ± 0.005 (*n* = 6); 7 weeks: Control 0.04 ± 0.003 (*n* = 6), BLZ945 0.04 ± 0.002 (*n* = 8)	4-week Control-BLZ945: *p *=* *0.0258, *t *=* *2.66, df = 27; 7-week Control-BLZ945: *p *=* *0.8450, *t *=* *0.522, df = 27; Control 4–7 weeks: *p *=* *0.2535, *t *=* *1.537, df = 27; BLZ945 4–7 weeks: *p *=* *0.8381, *t *=* *0.534, df = 27

Calyx of Held surface area (two-way ANOVA, Sidak’s multiple comparisons test)	3 weeks: Control 687.5 ± 35.70 (*n* = 4), BLZ945 680.1.8 ± 13.40 (*n* = 4); 4 weeks: Control 747.1 ± 21.19 (*n* = 5), BLZ945 707.8 ± 56.78 (*n* = 5)	3-week Control-BLZ945: *p *=* *0.9895, *t *=* *0.1310, df = 14; 4-week Control-BLZ945: *p *=* *0.7005, *t *=* *0.7724, df = 14; Control 3–4 weeks: *p *=* *0.4941, *t *=* *1.103, df = 14; BLZ945 3–4 weeks: *p *=* *0.8527, *t *=* *0.5126, df = 14

Polyinnervatioon of MNTB neurons (two-way ANOVA, Sidak’s multiple comparisons test)	3 weeks: Control 9.92 ± 6.75 (*n* = 4), BLZ945 38.78 ± 9.89 (*n* = 4); 4 weeks: Control 5.08 ± 3.15 (*n* = 5), BLZ945 16.59 ± 5.82 (*n* = 5)	3-week Control-BLZ945: *p *=* *0.0191, *t *=* *2.998, df = 14; 4-week Control-BLZ945: *p *=* *0.3645, *t *=* *1.336, df = 14; Control 3–4 weeks: *p *=* *0.8435, *t *=* *0.53, df = 14; BLZ945 3–4 weeks: *p *=* *0.0574, *t *=* *2.43, df = 14

Calyceal shape factor analysis (two-way ANOVA, Sidak’s multiple comparisons test)	3 weeks: Control 6.394 ± 0.16 (*n* = 4), BLZ945 5.6 ± 0.17 (*n* = 4); 4 weeks: Control 6.74 ± 0.13 (*n* = 5), BLZ945 6.35 ± 0.24 (*n* = 5)	3-week Control-BLZ945: *p *=* *0.3210, *t *=* *1.425, df = 14; 4-week Control-BLZ945: *p *=* *0.2488, *t *=* *1.594, df = 14; Control 3–4 weeks: *p *=* *0.3715, *t *=* *1.323, df = 14; BLZ945 3–4 weeks: *p *=* *0.3717, *t *=* *1.322, df = 14

**Table 4:** Two-way ANOVA and simple linear regressions

	Absolute peak latency (ms)								Simple linear regression			
Stimulus/ peak	Mean ± SEM		Treatment		Intensity		Interaction		Differences between slopes		Differences between elevations or intercepts	
	4 weeks	7 weeks	4 weeks	7 weeks	4 weeks	7 weeks	4 weeks	7 weeks	4 weeks	7 weeks	4 weeks	7 weeks

4 kHz peak I	Control 1.803 ± 0.035 (*n* = 13); BLZ945 2.011 ± 0.044 (*n* = 14)	Control 1.78 ± 0.04 (*n* = 11); BLZ945 1.93 ± 0.03 (*n* = 15)	*p* < 0.0001	*p* < 0.0001	*p* < 0.0001	*p* < 0.0001	*p* = 0.7740	*p* = 0.4004	*p* = 0.0410	*p* = 0.1317	Not possible to test	*p* < 0.0001
8 kHz peak I	Control 1.82 ± 0.036 (*n* = 13); BLZ945 1.94 ± 0.04 (*n* = 14)	Control 1.79 ± 0.04 (*n* = 11); BLZ945 1.87 ± 0.03 (*n* = 15)	*p* < 0.0001	*p* < 0.0001	*p* < 0.0001	*p* < 0.0001	*p* = 0.7755	*p* = 0.6425	*p* = 0.1907	*p* = 0.1613	*p* < 0.0001	*p* < 0.0001
12 kHz peak I	Control 1.88 ± 0.04 (*n* = 13); BLZ945 1.95 ± 0.04 (*n* = 14)	Control 1.80 ± 0.04 (*n* = 11); BLZ945 1.87 ± 0.03 (*n* = 15)	*p* < 0.0001	*p* < 0.0001	*p* < 0.0001	*p* < 0.0001	*p* = 0.7517	*p* = 0.9777	*p* = 0.3589	*p* = 0.1551	*p* < 0.0001	*p* < 0.0001
16 kHz peak I	Control 1.89 ± 0.04 (*n* = 13); BLZ945 1.93 ± 0.03 (*n* = 14)	Control 1.77 ± 0.03 (*n* = 11); BLZ945 1.86 ± 0.03 (*n* = 15)	*p* = 0.0020	*p* < 0.0001	*p* < 0.0001	*p* < 0.0001	*p* = 0.1362	*p* = 0.9942	*p* = 0.0021	*p* = 0.5215	Not possible to test	*p* < 0.0001
24 kHz peak I	Control 1.91 ± 0.04 (*n* = 13); BLZ945 1.80 ± 0.03 (*n* = 14)	Control 1.76 ± 0.03 (*n* = 11); BLZ945 1.81 ± 0.04 (*n* = 15)	*p* < 0.0001	*p* = 0.1081	*p* < 0.0001	*p* < 0.0001	*p* = 0.8522	*p* = 0.9943	*p* = 0.0728	*p* = 0.5176	*p* < 0.0001	*p* = 0.1525
32 kHz peak I	Control 1.91 ± 0.04 (*n* = 13); BLZ945 1.81 ± 0.03 (*n* = 14)	Control 1.83 ± 0.03 (*n* = 11); BLZ945 1.85 ± 0.03 (*n* = 15)	*p* = 0.0029	*p* = 0.5552	*p* = 0.0946	*p* = 0.1688	*p* = 0.9998	*p* = 0.9872	*p* = 0.8675	*p* = 0.8108	*p* = 0.0014	*p* = 0.6973
												
4 kHz peak II	Control 2.89 ± 0.04 (*n* = 13); BLZ945 3.18 ± 0.05 (*n* = 14)	Control 2.82 ± 0.04 (*n* = 11); BLZ945 2.99 ± 0.03 (*n* = 15)	*p* < 0.0001	*p* < 0.0001	*p* < 0.0001	*p* < 0.0001	*p* = 0.8598	*p* = 0.9661	*p* = 0.7283	*p* = 0.4145	*p* < 0.0001	*p* < 0.0001
8 kHz peak II	Control 3.05 ± 0.02 (*n* = 13); BLZ945 3.11 ± 0.04 (*n* = 14)	Control 2.88 ± 0.03 (*n* = 11); BLZ945 2.99 ± 0.04 (*n* = 15)	*p* = 0.0190	*p* = 0.0011	*p* < 0.0001	*p* < 0.0001	*p* = 0.5803	*p* > 0.9999	*p* = 0.6072	*p* = 0.8238	*p* = 0.0475	*p* = 0.0006
12 kHz peak II	Control 3.09 ± 0.04 (*n* = 13); BLZ945 3.17 ± 0.04 (*n* = 14)	Control 2.87 ± 0.04 (*n* = 11); BLZ945 2.95 ± 0.03 (*n* = 15)	*p* = 0.0003	*p* = 0.0334	*p* < 0.0001	*p* = 0.0002	*p* = 0.7647	*p* = 0.9898	*p* = 0.1824	*p* = 0.3472	*p* = 0.0003	*p* = 0.0089
16 kHz peak II	Control 3.05 ± 0.04 (*n* = 13); BLZ945 3.13 ± 0.03 (*n* = 14)	Control 2.83 ± 0.04 (*n* = 11); BLZ945 2.98 ± 0.04 (*n* = 15)	*p* = 0.0024	*p* < 0.0001	*p* < 0.0001	*p* < 0.0001	*p* = 0.9859	*p* = 0.9995	*p* = 0.3160	*p* = 0.7315	*p* = 0.0019	*p* < 0.0001
24 kHz peak II	Control 3.02 ± 0.06 (*n* = 13); BLZ945 2.76 ± 0.04 (*n* = 14)	Control 2.75 ± 0.02 (*n* = 11); BLZ945 2.67 ± 0.06 (*n* = 15)	*p* < 0.0001	*p* = 0.1407	*p* = 0.0040	*p* = 0.3100	*p* = 0.6485	*p* = 0.7841	*p* = 0.0431	*p* = 0.0567	Not possible to test	*p* = 0.0080
32 kHz peak II	Control 2.90 ± 0.09 (*n* = 13); BLZ945 2.72 ± 0.03 (*n* = 14)	Control 2.72 ± 0.03 (*n* = 11); BLZ945 2.67 ± 0.04 (*n* = 15)	*p* = 0.0006	*p* = 0.4970	*p* < 0.0001	*p* = 0.9996	*p* = 0.4457	*p* = 0.6959	*p* = 0.0042	*p* = 0.0507	Not possible to test	*p* = 0.0752
												
4 kHz peak III	Control 3.88 ± 0.04 (*n* = 13); BLZ945 4.19 ± 0.06 (*n* = 14)	Control 3.68 ± 0.03 (*n* = 11); BLZ945 3.76 ± 0.02 (*n* = 15)	*p* < 0.0001	*p* = 0.0641	*p* < 0.0001	*p* = 0.5772	*p* = 0.7320	*p* = 0.9884	*p* = 0.0285	*p* = 0.8082	Not possible to test	*p* = 0.0072
8 kHz peak III	Control 3.89 ± 0.03 (*n* = 13); BLZ945 4.12 ± 0.06 (*n* = 14)	Control 3.68 ± 0.02 (*n* = 11); BLZ945 3.78 ± 0.02 (*n* = 15)	*p* < 0.0001	*p* = 0.0179	*p* < 0.0001	*p* = 0.7081	*p* = 0.2898	*p* > 0.9999	*p* = 0.0109	*p* = 0.7434	Not possible to test	*p* = 0.0053
12 kHz peak III	Control 3.98 ± 0.04 (*n* = 13); BLZ945 4.21 ± 0.05 (*n* = 14)	Control 3.71 ± 0.02 (*n* = 11); BLZ945 3.82 ± 0.02 (*n* = 15)	*p* < 0.0001	*p* = 0.0113	*p* < 0.0001	*p* = 0.6296	*p* = 0.9039	*p* = 0.9884	*p* = 0.9176	*p* = 0.6784	*p* < 0.0001	*p* = 0.0018
16 kHz peak III	Control 4.02 ± 0.06 (*n* = 13); BLZ945 4.25 ± 0.04 (*n* = 14)	Control 3.72 ± 0.02 (*n* = 11); BLZ945 3.91 ± 0.03 (*n* = 15)	*p* < 0.0001	*p* < 0.0001	*p* < 0.0001	*p* = 0.6845	*p* = 0.8036	*p* = 0.9996	*p* = 0.0273	*p* = 0.7221	Not possible to test	*p* < 0.0001
24 kHz peak III	Control 3.98 ± 0.05 (*n* = 13); BLZ945 4.05 ± 0.03 (*n* = 14)	Control 3.77 ± 0.07 (*n* = 11); BLZ945 3.80 ± 0.05 (*n* = 15)	*p* = 0.1924	*p* = 0.7003	*p* = 0.0467	*p* = 0.1637	*p* = 0.9797	*p* = 0.9808	*p* = 0.2513	*p* = 0.7024	*p* = 0.1708	*p* = 0.4274
32 kHz peak III	Control 3.92 ± 0.09 (*n* = 13); BLZ945 4.07 ± 0.03 (*n* = 14)	Control 3.66 ± 0.04 (*n* = 11); BLZ945 3.88 ± 0.02 (*n* = 15)	*p* = 0.0060	*p* = 0.0114	*p* = 0.0001	*p* = 0.9482	*p* = 0.3418	*p* = 0.9991	*p* = 0.0023	*p* = 0.7152	Not possible to test	*p* = 0.0079
4 kHz peak IV	Control 4.88 ± 0.05 (*n* = 13); BLZ945 5.36 ± 0.07 (*n* = 14)	Control 4.62 ± 0.06 (*n* = 11); BLZ945 4.85 ± 0.04 (*n* = 15)	*p* < 0.0001	*p* < 0.0001	*p* < 0.0001	*p* = 0.0002	*p* = 0.9720	*p* = 0.5290	*p* = 0.1473	*p* = 0.5928	*p* < 0.0001	*p* < 0.0001
8 kHz peak IV	Control 4.92 ± 0.05 (*n* = 13); BLZ945 5.23 ± 0.06 (*n* = 14)	Control 4.57 ± 0.05 (*n* = 11); BLZ945 4.86 ± 0.03 (*n* = 15)	*p* < 0.0001	*p* < 0.0001	*p* < 0.0001	*p* = 0.0024	*p* = 0.3804	*p* = 0.9688	*p* = 0.6358	*p* = 0.2588	*p* < 0.0001	*p* < 0.0001
12 kHz peak IV	Control 4.96 ± 0.06 (*n* = 13); BLZ945 5.32 ± 0.08 (*n* = 14)	Control 4.62 ± 0.05 (*n* = 11); BLZ945 4.87 ± 0.04 (*n* = 15)	*p* < 0.0001	*p* < 0.0001	*p* < 0.0001	*p* = 0.0023	*p* = 0.9533	*p* = 0.9198	*p* = 0.3836	*p* = 0.7575	*p* < 0.0001	*p* < 0.0001
16 kHz peak IV	Control 4.93 ± 0.06 (*n* = 13); BLZ945 5.32 ± 0.06 (*n* = 14)	Control 4.62 ± 0.04 (*n* = 11); BLZ945 4.90 ± 0.04 (*n* = 15)	*p* < 0.0001	*p* < 0.0001	*p* < 0.0001	*p* = 0.0020	*p* = 0.9930	*p* = 0.9933	*p* = 0.6512	*p* = 0.9863	*p* < 0.0001	*p* < 0.0001
24 kHz peak IV	Control 5.01 ± 0.08 (*n* = 13); BLZ945 5.00 ± 0.03 (*n* = 14)	Control 4.66 ± 0.08 (*n* = 11); BLZ945 4.88 ± 0.03 (*n* = 15)	*p* = 0.8684	*p* = 0.0679	*p* = 0.0009	*p* = 0.2143	*p* = 0.6273	*p* = 0.9372	*p* = 0.0130	*p* = 0.2559	Not possible to test	*p* = 0.0048
32 kHz peak IV	Control 5.02 ± 0.13 (*n* = 13); BLZ945 5.10 ± 0.11 (*n* = 14)	Control 4.62 ± 0.05 (*n* = 11); BLZ945 4.83 ± 0.03 (*n* = 15)	*p* = 0.2854	*p* = 0.0060	*p* < 0.0001	*p* = 0.5935	*p* = 0.2226	*p* = 0.9938	*p* = 0.0027	*p* = 0.7382	Not possible to test	*p* = 0.0040
	Interpeak latency (ms)								Simple linear regression			
	Mean ± SEM		Treatment		Intensity		Interaction		Differences between slopes		Differences between elevations or intercepts	
Stimulus	4 weeks	7 weeks	4 weeks	7 weeks	4 weeks	7 weeks	4 weeks	7 weeks	4 weeks	7 weeks	4 weeks	7 weeks

4 kHz peak I–II	Control 1.04 ± 0.01 (*n* = 13); BLZ945 1.08 ± 0.02 (*n* = 14)	Control 1.05 ± 0.01 (*n* = 11); BLZ945 1.06 ± 0.01 (*n* = 15)	*p* = 0.0153	*p* = 0.5483	*p* = 0.9959	*p* = 0.9992	*p* = 0.8222	*p* = 0.9891	*p* = 0.2560	*p* = 0.9111	*p* = 0.0206	*p* = 0.6128
8 kHz peak I–II	Control 1.22 ± 0.02 (*n* = 13); BLZ945 1.17 ± 0.02 (*n* = 14)	Control 1.09 ± 0.01 (*n* = 11); BLZ945 1.11 ± 0.01 (*n* = 15)	*p* = 0.0140	*p* = 0.4202	*p* = 0.0731	*p* = 0.9810	*p* = 0.8531	*p* = 0.9632	*p* = 0.9154	*p* = 0.3888	*p* = 0.0028	*p* = 0.8857
12 kHz peak I–II	Control 1.22 ± 0.01 (*n* = 13); BLZ945 1.22 ± 0.02 (*n* = 14)	*n* =Control 1.09 ± 0.01 (*n* = 11); BLZ945 1.10 ± 0.01 (*n* = 15)	*p* = 0.9395	*p* = 0.6103	*p* = 0.3768	*p* = 0.9975	*p* = 0.9098	*p* = 0.9748	*p* = 0.6346	*p* = 0.8061	*p* = 0.7079	*p* = 0.8426
16 kHz peak I–II	Control 1.12 ± 0.08 (*n* = 13); BLZ945 1.15 ± 0.01 (*n* = 14)	Control 1.06 ± 0.01 (*n* = 11); BLZ945 1.12 ± 0.01 (*n* = 15)	*p* = 0.0653	*p* = 0.0671	*p* = 0.7001	*p* = 0.9954	*p* = 0.9939	*p* = 0.9911	*p* = 0.6778	*p* = 0.9407	*p* = 0.0770	*p* = 0.0770
24 kHz peak I–II	Control 1.06 ± 0.01 (*n* = 13); BLZ945 0.94 ± 0.02 (*n* = 14)	Control 0.99 ± 0.03 (*n* = 11); BLZ945 0.86 ± 0.02 (*n* = 15)	*p* = 0.0001	*p* = 0.0028	*p* = 0.8792	*p* = 0.9741	*p* = 0.3152	*p* = 0.5636	*p* = 0.6021	*p* = 0.0421	*p* < 0.0001	Not possible to test
32 kHz peak I–II	Control 0.92 ± 0.04 (*n* = 13); BLZ945 0.91 ± 0.01 (*n* = 14)	Control 0.89 ± 0.04 (*n* = 11); BLZ945 0.82 ± 0.01 (*n* = 15)	*p* = 0.7968	*p* = 0.0893	*p* = 0.2994	*p* = 0.6474	*p* = 0.3764	*p* = 0.1604	*p* = 0.0067	*p* = 0.0030	Not possible to test	Not possible to test
												
4 kHz peak II–III	Control 0.99 ± 0.02 (*n* = 13); BLZ945 1.00 ± 0.03 (*n* = 14)	Control 0.83 ± 0.04 (*n* = 11); BLZ945 0.76 ± 0.02 (*n* = 15)	*p* = 0.7107	*p* = 0.0339	*p* = 0.1573	*p* = 0.0309	*p* = 0.6656	*p* = 0.7258	*p* = 0.0021	*p* = 0.1644	Not possible to test	*p* = 0.0079
8 kHz peak II–III	Control 0.84 ± 0.01 (*n* = 13); BLZ945 1.01 ± 0.03 (*n* = 14)	Control 0.81 ± 0.02 (*n* = 11); BLZ945 0.79 ± 0.03 (*n* = 15)	*p* < 0.0001	*p* = 0.6447	*p* = 0.0426	*p* = 0.0143	*p* = 0.6052	*p* = 0.9630	*p* = 0.0179	*p* = 0.8336	Not possible to test	*p* = 0.9809
12 kHz peak II–III	Control 0.89 ± 0.01 (*n* = 13); BLZ945 1.04 ± 0.02 (*n* = 14)	Control 0.83 ± 0.03 (*n* = 11); BLZ945 0.85 ± 0.02 (*n* = 15)	*p* < 0.0001	*p* = 0.4802	*p* = 0.1744	*p* = 0.0359	*p* = 0.7669	*p* = 0.8068	*p* = 0.1599	*p* = 0.9206	*p* < 0.0001	*p* = 0.2105
16 kHz peak II–III	Control 0.97 ± 0.03 (*n* = 13); BLZ945 1.13 ± 0.01 (*n* = 14)	Control 0.89 ± 0.03 (*n* = 11); BLZ945 0.93 ± 0.02 (*n* = 15)	*p* < 0.0001	*p* = 0.1727	*p* = 0.3229	*p* = 0.0254	*p* = 0.8809	*p* = 0.9121	*p* = 0.0957	*p* = 0.8282	*p* < 0.0001	*p* = 0.1373
24 kHz peak II–III	Control 1.01 ± 0.02 (*n* = 13); BLZ945 1.32 ± 0.02 (*n* = 14)	Control 1.02 ± 0.06 (*n* = 11); BLZ945 1.03 ± 0.03 (*n* = 15)	*p* < 0.0001	*p* = 0.0306	*p* = 0.8895	*p* = 0.7698	*p* = 0.8571	*p* = 0.1234	*p* = 0.8095	*p* = 0.0101	*p* < 0.0001	Not possible to test
32 kHz peak II–III	Control 1.02 ± 0.02 (*n* = 13); BLZ945 1.35 ± 0.02 (*n* = 14)	Control 0.94 ± 0.03 (*n* = 11); BLZ945 1.20 ± 0.05 (*n* = 14)	*p* < 0.0001	*p* < 0.0001	*p* = 0.7796	*p* = 0.4237	*p* = 0.7163	*p* = 0.6276	*p* = 0.6767	*p* = 0.0622	*p* < 0.0001	*p* < 0.0001
4 kHz peak III–IV	Control 1.00 ± 0.025 (*n* = 13); BLZ945 1.19 ± 0.02 (*n* = 14)	Control 0.94 ± 0.03 (*n* = 11); BLZ945 1.09 ± 0.02 (*n* = 15)	*p* < 0.0001	*p* < 0.0001	*p* = 0.3084	*p* = 0.0016	*p* = 0.9181	*p* = 0.6572	*p* = 0.8436	*p* = 0.2525	*p* < 0.0001	*p* < 0.0001
8 kHz peak III–IV	Control 1.04 ± 0.02 (*n* = 13); BLZ945 1.11 ± 0.02 (*n* = 14)	Control 0.88 ± 0.04 (*n* = 11); BLZ945 1.09 ± 0.02 (*n* = 15)	*p* = 0.0007	*p* < 0.0001	*p* = 0.1212	*p* = 0.0039	*p* = 0.0468	*p* = 0.9099	*p* = 0.0002	*p* = 0.0554	Not possible to test	*p* < 0.0001
12 kHz peak III–IV	Control 0.99 ± 0.02 (*n* = 13); BLZ945 1.13 ± 0.03 (*n* = 14)	Control 0.91 ± 0.03 (*n* = 11); BLZ945 1.05 ± 0.03 (*n* = 15)	*p* < 0.0001	*p* < 0.0001	*p* < 0.0001	*p* = 0.0006	*p* = 0.7583	*p* = 0.8825	*p* = 0.1432	*p* = 0.8958	*p* < 0.0001	*p* < 0.0001
16 kHz peak III–IV	Control 0.95 ± 0.01 (*n* = 13); BLZ945 1.09 ± 0.03 (*n* = 14)	Control 0.90 ± 0.03 (*n* = 11); BLZ945 1.00 ± 0.02 (*n* = 15)	*p* < 0.0001	*p* = 0.0006	*p* = 0.0133	*p* = 0.0011	*p* = 0.6417	*p* = 0.4585	*p* = 0.0465	*p* = 0.3260	Not possible to test	*p* < 0.0001
24 kHz peak III–IV	Control 1.02 ± 0.37 (*n* = 13); BLZ945 0.95 ± 0.011 (*n* = 14)	Control 0.89 ± 0.02 (*n* = 11); BLZ945 1.00 ± 0.03 (*n* = 15)	*p* = 0.0333	*p* = 0.3942	*p* = 0.2306	*p* = 0.9896	*p* = 0.5691	*p* = 0.9452	*p* = 0.0593	*p* = 0.4044	*p* = 0.0446	*p* = 0.2133
32 kHz peak III–IV	Control 1.02 ± 0.03 (*n* = 13); BLZ945 0.94 ± 0.02 (*n* = 14)	Control 0.92 ± 0.04 (*n* = 11); BLZ945 0.95 ± 0.03 (*n* = 15)	*p* = 0.0702	*p* = 0.4964	*p* = 0.5917	*p* = 0.0305	*p* = 0.8558	*p* = 0.7748	*p* = 0.4193	*p* = 0.9671	*p* = 0.0885	*p* = 0.4816
												
4 kHz peak I–III	Control 2.08 ± 0.02 (*n* = 13); BLZ945 2.17 ± 0.02 (*n* = 14)	Control 1.88 ± 0.03 (*n* = 11); BLZ945 1.82 ± 0.01 (*n* = 15)	*p* = 0.0017	*p* = 0.1841	*p* = 0.8207	*p* = 0.3167	*p* = 0.9195	*p* = 0.9957	*p* = 0.1090	*p* = 0.2727	*p* = 0.0012	*p* = 0.0614
8 kHz peak I–III	Control 2.06 ± 0.01 (*n* = 13); BLZ945 2.18 ± 0.02 (*n* = 14)	Control 1.89 ± 0.03 (*n* = 11); BLZ945 1.90 ± 0.02 (*n* = 15)	*p* < 0.0001	*p* = 0.7250	*p* = 0.9903	*p* = 0.0591	*p* = 0.4979	*p* = 0.9992	*p* = 0.0308	*p* = 0.3458	Not possible to test	*p* = 0.9125
12 kHz peak I–III	Control 2.10 ± 0.01 (*n* = 13); BLZ945 2.26 ± 0.01 (*n* = 14)	Control 1.92 ± 0.03 (*n* = 11); BLZ945 1.95 ± 0.02 (*n* = 15)	*p* < 0.0001	*p* = 0.3173	*p* = 0.9978	*p* = 0.1046	*p* = 0.9658	*p* = 0.9686	*p* = 0.5763	*p* = 0.9010	*p* < 0.0001	*p* = 0.2369
16 kHz peak I–III	Control 2.12 ± 0.02 (*n* = 13); BLZ945 2.32 ± 0.01 (*n* = 14)	Control 1.94 ± 0.03 (*n* = 11); BLZ945 2.05 ± 0.02 (*n* = 15)	*p* < 0.0001	*p* = 0.0096	*p* = 0.9716	*p* = 0.3775	*p* = 0.8827	*p* = 0.9947	*p* = 0.1650	*p* = 0.9090	*p* < 0.0001	*p* = 0.0076
24 kHz peak I–III	Control 2.07 ± 0.02 (*n* = 13); BLZ945 2.26 ± 0.01 (*n* = 14)	Control 2.01 ± 0.05 (*n* = 11); BLZ945 1.99 ± 0.02 (*n* = 15)	*p* < 0.0001	*p* = 0.7448	*p* = 0.9949	*p* = 0.8627	*p* = 0.9725	*p* = 0.8470	*p* = 0.5676	*p* = 0.4364	*p* < 0.0001	*p* = 0.7944
32 kHz peak I–III	Control 1.94 ± 0.04 (*n* = 13); BLZ945 2.25 ± 0.02 (*n* = 14)	Control 1.83 ± 0.05 (*n* = 11); BLZ945 2.03 ± 0.04 (*n* = 15)	*p* < 0.0001	*p* = 0.0028	*p* = 0.3596	*p* = 0.4304	*p* = 0.2410	*p* = 0.9929	*p* = 0.0126	*p* = 0.7414	Not possible to test	*p* = 0.0012
												
4 kHz peak I–IV	Control 3.09 ± 0.02 (*n* = 13); BLZ945 3.37 ± 0.03 (*n* = 15)	Control 2.84 ± 0.04 (*n* = 11); BLZ945 2.92 ± 0.02 (n = 15)	*n* =*p* < 0.0001	*p* = 0.0431	*p* = 0.0020	*p* = 0.7609	*p* = 0.9895	*p* = 0.7424	*p* = 0.3397	*p* = 0.9785	*p* < 0.0001	*p* = 0.0021
8 kHz peak I–IV	Control 3.10 ± 0.02 (*n* = 13); BLZ945 3.30 ± 0.03 (*n* = 14)	Control 2.78 ± 0.02 (*n* = 11); BLZ945 2.99 ± 0.01 (*n* = 15)	*p* < 0.0001	*p* < 0.0001	*p* = 0.1157	*p* = 0.9974	*p* = 0.2013	*p* = 0.9861	*p* = 0.3003	*p* = 0.4020	*p* < 0.0001	*p* < 0.0001
12 kHz peak I–II	Control 3.10 ± 0.02 (*n* = 13); BLZ945 3.39 ± 0.04 (*n* = 14)	Control 2.83 ± 0.03 (*n* = 11); BLZ945 2.97 ± 0.03 (*n* = 15)	*p* < 0.0001	*p* = 0.0269	*p* < 0.0001	*p* = 0.8547	*p* = 0.9535	*p* = 0.9391	*p* = 0.2057	*p* = 0.8016	*p* < 0.0001	*p* = 0.0265
16 kHz peak I–IV	Control 3.06 ± 0.03 (*n* = 13); BLZ945 3.41 ± 0.03 (*n* = 14)	Control 2.85 ± 0.02 (*n* = 11); BLZ945 3.04 ± 0.02 (*n* = 15)	*p* < 0.0001	*p* < 0.0001	*p* = 0.0216	*p* = 0.8560	*p* = 0.9811	*p* = 0.9787	*p* = 0.6924	*p* = 0.8216	*p* < 0.0001	*p* < 0.0001
24 kHz peak I–IV	Control 3.09 ± 0.04 (*n* = 13); BLZ945 3.20 ± 0.02 (*n* = 14)	Control 2.90 ± 0.06 (*n* = 11); BLZ945 2.99 ± 0.02 (*n* = 15)	*p* = 0.0203	*p* = 0.1345	*p* = 0.3332	*p* = 0.8446	*p* = 0.6537	*p* = 0.5978	*p* = 0.0266	*p* = 0.0856	Not possible to test	*p* = 0.0052
32 kHz peak I–IV	Control 2.96 ± 0.06 (*n* = 13); BLZ945 3.18 ± 0.03 (*n* = 14)	Control 2.76 ± 0.03 (*n* = 11); BLZ945 2.98 ± 0.03 (*n* = 15)	*p* < 0.0001	*p* = 0.0006	*p* = 0.0623	*p* = 0.6208	*p* = 0.1412	*p* = 0.9914	*p* = 0.0030	*p* = 0.7591	Not possible to test	*p* = 0.0002
	Trough-peak amplitude (μV) (ms)								Simple linearregression			
	Mean ± SEM		Treatment		Intensity		Interaction		Differences between slopes		Differences between elevations or intercepts	
Stimulus	4 weeks	7 weeks	4 weeks	7 weeks	4 weeks	7 weeks	4 weeks	7 weeks	4 weeks	7 weeks	4 weeks	7 weeks

4 kHz peak I	Control 2.44 ± 0.33 (*n* = 13); BLZ945 2.19 ± 0.43 (*n* = 14)	Control 1.18 ± 0.13 (*n* = 11); BLZ945 1.04 ± 0.14 (*n* = 15)	*p* = 0.0321	*p* = 0.2298	*p* < 0.0001	*p* < 0.0001	*p* = 0.4344	*p* > 0.9999	*p* = 0.0003	*p* = 0.7428	Not possible to test	*p* = 0.1463
8 kHz peak I	Control 2.35 ± 0.40 (*n* = 13); BLZ945 2.14 ± 0.40 (*n* = 14)	Control 1.05 ± 0.14 (*n* = 11); BLZ945 0.94 ± 0.14 (*n* = 15)	*p* < 0.0001	*p* = 0.2580	*p* < 0.0001	*p* < 0.0001	*p* = 0.9867	*p* = 0.9896	*p* = 0.6324	*p* = 0.5651	*p* = 0.0219	*p* = 0.1100
12 kHz peak I	Control 2.28 ± 0.40 (*n* = 13); BLZ945 1.78 ± 0.35 (*n* = 14)	Control 1.01 ± 0.12 (*n* = 11); BLZ945 0.96 ± 0.15 (*n* = 15)	*p* < 0.0001	*p* = 0.5704	*p* < 0.0001	*p* < 0.0001	*p* = 0.9867	*p* = 0.9970	*p* = 0.6766	*p* = 0.2858	*p* < 0.0001	*p* = 0.6782
16 kHz peak I	Control 2.12 ± 0.36 (*n* = 13); BLZ945 1.80 ± 0.33 (*n* = 14)	Control 0.95 ± 0.14 (*n* = 11); BLZ945 0.91 ± 0.12 (*n* = 15)	*p* = 0.0002	*p* = 0.6791	*p* < 0.0001	*p* < 0.0001	*p* = 0.9739	*p* = 0.9987	*p* = 0.4497	*p* = 0.3577	*p* < 0.0001	*p* = 0.4971
24 kHz peak I	Control 1.57 ± 0.32 (*n* = 13); BLZ945 1.40 ± 0.28 (*n* = 14)	Control 0.80 ± 0.13 (*n* = 11); BLZ945 0.75 ± 0.11 (*n* = 15)	*p* = 0.0220	*p* = 0.5020	*p* < 0.0001	*p* < 0.0001	*p* = 0.8737	*p* = 0.9967	*p* = 0.0933	*p* = 0.6140	*p* = 0.0164	*p* = 0.3243
32 kHz peak I	Control 1.22 ± 0.26 (*n* = 13); BLZ945 1.21 ± 0.23 (*n* = 14)	Control 0.42 ± 0.09 (*n* = 11); BLZ945 0.77 ± 0.09 (*n* = 15)	*p* = 0.9183	*p* = 0.7245	*p* < 0.0001	*p* < 0.0001	*p* = 0.8965	*p* = 0.9998	*p* = 0.4540	*p* = 0.9961	*p* = 0.6648	*p* = 0.6956
												
4 kHz peak II	Control 3.18 ± 0.26 (*n* = 13); BLZ945 2.89 ± 0.54 (*n* = 14)	Control 2.34 ± 0.25 (*n* = 11); BLZ945 2.31 ± 0.18 (*n* = 15)	*p* = 0.1470	*p* = 0.8947	*p* < 0.0001	*p* < 0.0001	*p* = 0.0065	*p* = 0.8911	*p* < 0.0001	*p* = 0.1516	Not possible to test	*p* = 0.3248
8 kHz peak II	Control 3.79 ± 0.43 (*n* = 13); BLZ945 2.80 ± 0.49 (*n* = 14)	Control 2.44 ± 0.30 (*n* = 11); BLZ945 2.19 ± 0.21 (*n* = 15)	*p* < 0.0001	*p* = 0.1876	*p* < 0.0001	*p* < 0.0001	*p* = 0.5675	*p* = 0.9756	*p* = 0.9709	*p* = 0.1704	*p* < 0.0001	*p* = 0.0174
12 kHz peak II	Control 3.68 ± 0.42 (*n* = 13); BLZ945 2.51 ± 0.47 (*n* = 14)	Control 2.46 ± 0.29 (*n* = 11); BLZ945 2.25 ± 0.29 (*n* = 15)	*p* < 0.0001	*p* = 0.2617	*p* < 0.0001	*p* < 0.0001	*p* = 0.0448	*p* = 0.9824	*p* = 0.0264	*p* = 0.9547		*p* = 0.0847
16 kHz peak II	Control 3.57 ± 0.43 (*n* = 13); BLZ945 2.59 ± 0.42 (*n* = 14)	Control 2.44 ± 0.32 (*n* = 11); BLZ945 2.28 ± 0.21 (*n* = 15)	*p* < 0.0001	*p* = 0.3469	*p* < 0.0001	*p* < 0.0001	*p* = 0.9849	*p* = 0.6964	*p* = 0.4983	*p* = 0.0200	*p* < 0.0001	Not possible to test
24 kHz peak II	Control 1.90 ± 0.18 (*n* = 13); BLZ945 1.61 ± 0.11 (*n* = 14)	Control 1.31 ± 0.15 (*n* = 11); BLZ945 1.22 ± 0.03 (*n* = 15)	*p* = 0.0053	*p* = 0.4828	*p* < 0.0001	*p* = 0.2868	*p* = 0.1501	*p* = 0.2803	*p* = 0.0097	*p* = 0.0010	Not possible to test	Not possible to test
32 kHz peak II	Control 1.41 ± 0.10 (*n* = 13); BLZ945 1.30 ± 0.10 (*n* = 14)	Control 1.23 ± 0.09 (*n* = 11); BLZ945 1.18 ± 0.04 (*n* = 15)	*p* = 0.5935	*p* = 0.5935	*p* = 0.0048	*p* = 0.8206	*p* = 0.6878	*p* = 0.5643	*p* = 0.0503	*p* = 0.0184	*p* = 0.4283	Not possible to test
												
4 kHz peak III	Control 2.71 ± 0.36 (*n* = 13); BLZ945 1.83 ± 0.30 (*n* = 14)	Control 1.54 ± 0.25 (*n* = 11); BLZ945 1.60 ± 0.23 (*n* = 15)	*p* < 0.0001	*p* = 0.5893	*p* < 0.0001	*p* < 0.0001	*p* = 0.9414	*p* = 0.6150	*p* = 0.4075	*p* = 0.9775	*p* < 0.0001	*p* = 0.9607
8 kHz peak III	Control 2.87 ± 0.40 (*n* = 13); BLZ945 2.16 ± 0.33 (*n* = 14)	Control 1.96 ± 0.32 (*n* = 11); BLZ945 1.87 ± 0.25 (*n* = 15)	*p* < 0.0001	*p* = 0.4410	*p* < 0.0001	*p* < 0.0001	*p* = 0.9013	*p* = 0.6888	*p* = 0.3396	*p* = 0.0894	*p* < 0.0001	*p* = 0.0641
12 kHz peak III	Control 2.46 ± 0.39 (*n* = 13); BLZ945 1.75 ± 0.34 (*n* = 14)	Control 1.70 ± 0.29 (*n* = 11); BLZ945 1.65 ± 0.23 (*n* = 15)	*p* < 0.0001	*p* = 0.6165	*p* < 0.0001	*p* < 0.0001	*p* = 0.0003	*p* = 0.7180	*p* = 0.7803	*p* = 0.1604	*p* < 0.0001	*p* = 0.1493
16 kHz peak III	Control 2.37 ± 0.38 (*n* = 13); BLZ945 1.71 ± 0.29 (*n* = 14)	Control 1.72 ± 0.26 (*n* = 11); BLZ945 1.68 ± 0.22 (*n* = 15)	*p* < 0.0001	*p* = 0.7426	*p* < 0.0001	*p* < 0.0001	*p* = 0.0152	*p* = 0.8593	*p* = 0.0539	*p* = 0.2221	*p* < 0.0001	*p* = 0.5628
24 kHz peak III	Control 2.19 ± 0.28 (*n* = 13); BLZ945 2.07 ± 0.30 (*n* = 14)	Control 1.80 ± 0.17 (*n* = 11); BLZ945 1.77 ± 0.22 (*n* = 15)	*p* = 0.2898	*p* = 0.8442	*p* < 0.0001	*p* < 0.0001	*p* = 0.9822	*p* = 0.8618	*p* = 0.6517	*p* = 0.1920	*p* = 0.2912	*p* = 0.8913
32 kHz peak III	Control 1.89 ± 0.28 (*n* = 13); BLZ945 2.07 ± 0.27 (*n* = 14)	Control 1.45 ± 0.16 (*n* = 11); BLZ945 1.74 ± 0.15 (*n* = 15)	*p* = 0.0895	*p* = 0.0329	*p* < 0.0001	*p* < 0.0001	*p* = 0.9441	*p* = 0.9858	*p* = 0.9026	*p* = 0.8422	*p* = 0.0880	*p* = 0.0143
4 kHz peak IV	Control 1.91 ± 0.10 (*n* = 13); BLZ945 1.38 ± 0.11 (*n* = 14)	Control 1.21 ± 0.05 (*n* = 11); BLZ945 1.28 ± 0.04 (*n* = 15)	*p* < 0.0001	*p* = 0.4991	*p* = 0.0004	*p* = 0.9954	*p* = 0.7772	*p* = 0.9608	*p* = 0.1818	*p* = 0.5307	*p* < 0.0001	*p* = 0.5992
8 kHz peak IV	Control 1.34 ± 0.12 (*n* = 13); BLZ945 1.41 ± 0.10 (*n* = 14)	Control 1.26 ± 0.04 (*n* = 11); 1.22 ± 0.04 (*n* = 15)	*p* < 0.0001	*p* = 0.6839	*p* < 0.0001	*p* = 0.9947	*p* = 0.3158	*p* = 0.9613	*p* = 0.3919	*p* = 0.2533	*p* < 0.0001	*p* = 0.8604
12 kHz peak IV	Control 1.75 ± 0.10 (*n* = 13); BLZ945 1.22 ± 0.12 (*n* = 14)	Control 1.16 ± 0.05 (*n* = 11); BLZ945 1.10 ± 0.03 (*n* = 15)	*p* < 0.0001	*p* = 0.5629	*p* < 0.0001	*p* = 0.9520	*p* = 0.0363	*p* = 0.9867	*p* = 0.0261	*p* = 0.5088	Not possible to test	*p* = 0.2207
16 kHz peak IV	Control 1.98 ± 0.18 (*n* = 13); BLZ945 1.28 ± 0.13 (*n* = 14)	Control 1.35 ± 0.04 (*n* = 11); BLZ945 1.11 ± 0.03 (*n* = 15)	*p* < 0.0001	*p* = 0.0157	*p* < 0.0001	*p* = 0.9762	*p* = 0.2837	*p* = 0.9966	*p* = 0.8223	*p* = 0.6633	*p* < 0.0001	*p* = 0.0072
24 kHz peak IV	Control 1.76 ± 0.14 (*n* = 13); BLZ945 1.30 ± 0.11 (*n* = 14)	Control 1.12 ± 0.07 (*n* = 11); BLZ945 1.08 ± 0.07 (*n* = 15)	*p* < 0.0001	*p* = 0.7217	*p* < 0.0001	*p* = 0.1788	*p* = 0.8879	*p* = 0.9796	*p* = 0.2887	*p* = 0.7785	*p* < 0.0001	*p* = 0.5245
32 kHz peak IV	Control 1.85 ± 0.16 (*n* = 13); BLZ945 1.53 ± 0.14 (*n* = 14)	Control 1.16 ± 0.08 (*n* = 11); BLZ945 1.26 ± 0.04(*n* = 15)	*p* = 0.0076	*p* = 0.3972	*p* < 0.0001	*p* = 0.9848	*p* = 0.9497	*p* = 0.5497	*p* = 0.4391	*p* = 0.8618	*p* = 0.0031	*p* = 0.6898

**Table 5:** Sidak’s multiple comparisons

		Absolute peak latency (ms)											
Peak I	Frequency (kHz)	4		8		12		16		24		32	
		4 weeks	7 weeks	4 weeks	7 weeks	4 weeks	7 weeks	4 weeks	7 weeks	4 weeks	7 weeks	4 weeks	7 weeks
Intensity (dB SPL)	80	<0.0001	0.0154	0.142	0.0704	0.9144	0.073	0.0148	0.9989	0.971	>0.9999	0.9719	>0.9999
	75	<0.0001	0.0161	0.0107	0.6141	0.3324	0.8812	0.0164	0.3791	0.9382	>0.9999	0.8797	>0.9999
	70	0.0002	0.0035	0.0063	0.1509	0.3323	0.6701	0.7302	0.5615	0.6526	>0.9999	0.9902	>0.9999
	65	0.0005	0.0002	0.004	0.015	0.0062	0.3586	0.7599	0.7856	0.8392	0.9998	0.9781	>0.9999
	60	<0.0001	0.0001	0.0819	0.041	0.1472	0.3005	>0.9999	0.3754	0.0982	0.996	0.8802	>0.9999
	55	<0.0001	0.0002	0.1598	0.0687	0.4281	0.2698	>0.9999	0.6131	0.5943	>0.9999	0.9438	0.9891
	50	<0.0001	0.0003	0.0517	0.076	0.9968	0.3716	>0.9999	0.2265	0.4666	0.9915	0.6895	>0.9999
	45	<0.0001	0.0003	0.0001	0.0235	0.9997	0.9985	>0.9999	0.1687	0.1566	>0.9999	0.9761	>0.9999
	40	<0.0001	0.0569	0.0004	0.3129	>0.9999	0.9915	>0.9999	0.616	0.0441	0.9885	0.9999	0.9919
	35	<0.0001	0.6835	0.0591	>0.9999	0.5309	>0.9999	>0.9999	0.8837		0.9894		
	30	0.0027	>0.9999		>0.9999	0.2878	>0.9999	0.9738	0.6585				
	25		0.9999		>0.9999	0.739	>0.9999	>0.9999	0.9991				
	20		0.995		>0.9999	>0.9999	>0.9999						
	15		0.9948				0.9977						
	10												
Peak II	Frequency (kHz)	4		8		12		16		24		32	
		4 weeks	7 weeks	4 weeks	7 weeks	4 weeks	7 weeks	4 weeks	7 weeks	4 weeks	7 weeks	4 weeks	7 weeks
Intensity (dB SPL)	80	0.015	0.5411	0.9932	0.9179	0.9844	0.9554	0.7955	0.9297	>0.9999	0.9601	>0.9999	0.9604
	75	0.0829	0.623	0.9826	0.9494	0.8154	0.9389	0.8754	0.9598	0.7029	0.7113	0.9999	0.9616
	70	0.0629	0.8672	0.9968	0.9987	0.1797	0.9887	0.9518	0.9694	0.2931	0.7088	0.9784	0.6067
	65	0.3147	0.4749	0.9956	>0.9999	0.6802	0.9929	0.8351	0.8741	0.1661	0.802	0.9994	0.93
	60	0.7408	0.0855	>0.9999	0.9996	0.3242	>0.9999	0.9547	0.9938	0.1172	0.9022	0.9941	0.9996
	55	0.9414	0.0574	0.9749	0.999	>0.9999	0.9772	0.9981	0.6225	0.1616	0.9837	0.9287	0.9998
	50	0.885	0.6198	>0.9999	0.8977	>0.9999	>0.9999	>0.9999	0.9004	0.8599	0.9996	0.7632	>0.9999
	45	0.0736	0.7625	0.8146	0.9982	>0.9999	>0.9999	>0.9999	0.9991	0.6202	>0.9999	0.4334	0.9974
	40	0.1948	>0.9999	0.7421	>0.9999	>0.9999	0.9819	>0.9999	>0.9999	0.4782	0.9989	0.0707	0.9294
	35	0.3042	0.9998	0.5336	0.998	>0.9999	0.9998	0.9978	0.9612	0.3795	0.9992		
	30	0.2715	>0.9999		>0.9999	>0.9999	>0.9999	>0.9999	0.814				
	25		>0.9999		0.9958	0.9984	>0.9999	0.9715	0.9212				
	20		0.9924		0.9633	0.7481	0.9998						
	15		0.997										
	10												
Peak III	Frequency (kHz)	4		8		12		16		24		32	
		4 weeks	7 weeks	4 weeks	7 weeks	4 weeks	7 weeks	4 weeks	7 weeks	4 weeks	7 weeks	4 weeks	7 weeks
Intensity (dB SPL)	80	0.0892	>0.9999	0.2589	0.9997	0.6618	0.9508	0.0869	0.6097	0.7121	>0.9999	0.0326	0.9975
	75	0.0931	>0.9999	0.7414	0.9999	0.0564	0.9542	0.0339	0.972	0.9859	>0.9999	0.4689	0.825
	70	0.1659	>0.9999	0.1266	>0.9999	0.0024	0.9981	0.0523	0.9129	>0.9999	>0.9999	0.3291	0.9981
	65	0.2881	0.9999	0.1423	0.9998	0.0101	>0.9999	0.0934	0.9992	>0.9999	0.9997	0.5173	0.989
	60	0.5546	0.9817	0.4306	0.9383	0.3956	>0.9999	0.2073	0.9989	>0.9999	0.9995	0.9985	0.9686
	55	0.3345	0.922	0.3093	0.9867	0.5178	0.8474	0.3623	0.9216	>0.9999	>0.9999	>0.9999	0.9907
	50	0.1898	0.9493	0.1087	0.9832	0.6383	0.7323	0.7939	0.9927	>0.9999	>0.9999	0.9995	0.9991
	45	0.0022	0.9873	0.008	>0.9999	0.6042	0.9886	0.8632	0.6164	>0.9999	0.9565	0.9992	0.9993
	40	0.0015	0.9906	0.0013	0.988	0.2169	0.9999	0.9932	0.8396	>0.9999	0.9997	>0.9999	0.9031
	35	0.0324	>0.9999	0.0005	>0.9999	0.3112	>0.9999	0.1275	0.9472		>0.9999		
	30	0.113	>0.9999		>0.9999	0.095	>0.9999	>0.9999	0.9577				
	25		>0.9999		>0.9999	0.0471	>0.9999	>0.9999	0.9943				
	20		0.9954		0.9998	0.7571	0.9989						
	15		>0.9999				0.9998						
	10												
Peak IV	Frequency (kHz)	4		8		12		16		24		32	
		4 weeks	7 weeks	4 weeks	7 weeks	4 weeks	7 weeks	4 weeks	7 weeks	4 weeks	7 weeks	4 weeks	7 weeks
Intensity (dB SPL)	80	0.002	0.9658	0.0093	0.4017	0.0385	0.4587	0.0096	0.4029	0.8486	0.8909	0.6344	0.9758
	75	0.0007	0.8294	0.0146	0.0402	0.0456	0.7979	0.0106	0.7975	0.9672	0.946	0.98	0.8348
	70	0.0014	0.3602	0.0013	0.0924	0.0135	0.6328	0.0274	0.1067	>0.9999	0.9875	0.4351	0.976
	65	0.0022	0.2856	0.0025	0.079	0.0131	0.8566	0.0554	0.9267	>0.9999	0.9856	0.4067	0.9011
	60	0.0014	0.0079	0.0901	0.0881	0.0488	0.9458	0.0369	0.8932	>0.9999	0.3691	>0.9999	>0.9999
	55	0.0006	0.2969	0.8889	0.1695	0.0777	0.1033	0.0248	0.4111	>0.9999	0.9782	>0.9999	0.9987
	50	<0.0001	0.1709	0.4232	0.0626	0.0244	0.2693	0.4096	0.179	0.9968	0.9961	>0.9999	0.9997
	45	<0.0001	0.0244	0.115	0.4409	0.1032	0.6082	0.0112	0.4635	0.8842	0.9997	0.8844	0.9483
	40	0.0004	0.5158	0.0035	0.2362	0.0416	0.9972	0.2211	0.239	0.9608	>0.9999	0.8327	0.8375
	35	0.0087	>0.9999	0.0073	>0.9999	0.0621	0.9932	0.0177	0.9993		0.9996	0.9989	
	30		>0.9999		0.9999	0.0005	>0.9999	0.7953	0.9605				
	25		0.9782		0.9988	0.0238	>0.9999		0.9844				
	20		0.9951		0.9606		0.9164						
	15		>0.9999				0.3562						
	10												
		Interpeak Latency (ms)											
Peak I–II	Frequency (kHz)	4		8		12		16		24		32	
		4 weeks	7 weeks	4 weeks	7 weeks	4 weeks	7 weeks	4 weeks	7 weeks	4 weeks	7 weeks	4 weeks	7 weeks
Intensity (dB SPL)	80	0.5664	>0.9999	>0.9999	>0.9999	>0.9999	>0.9999	>0.9999	0.9849	0.977	0.7201	0.462	0.4269
	75	0.8981	>0.9999	0.9996	>0.9999	>0.9999	0.9999	>0.9999	>0.9999	0.6867	0.2952	0.9813	0.4294
	70	0.747	>0.9999	0.9902	>0.9999	>0.9999	>0.9999	>0.9999	>0.9999	0.3337	0.1715	>0.9999	0.0423
	65	0.9919	>0.9999	0.9878	0.9996	>0.9999	>0.9999	0.9975	0.9983	0.0929	0.214	>0.9999	0.2511
	60	>0.9999	>0.9999	0.6939	>0.9999	0.9988	>0.9999	0.9644	>0.9999	0.3849	0.2568	0.9999	0.9934
	55	>0.9999	0.9982	0.19	>0.9999	0.9989	>0.9999	0.9936	0.9765	0.1609	0.9301	>0.9999	>0.9999
	50	>0.9999	>0.9999	0.8245	>0.9999	>0.9999	>0.9999	>0.9999	>0.9999	0.9967	0.8362	>0.9999	>0.9999
	45	0.997	>0.9999	0.9989	>0.9999	>0.9999	>0.9999	>0.9999	>0.9999	0.9883	>0.9999	0.6801	0.9948
	40	0.9996	0.9537	>0.9999	>0.9999	>0.9999	0.9999	>0.9999	>0.9999	0.9928	>0.9999	0.8977	0.9455
	35	0.9997	>0.9999	0.9998	0.9994	0.9924	0.9928	0.9999	0.9998		>0.9999		
	30	0.9546	>0.9999		>0.9999	0.9969	>0.9999	>0.9999	0.9975				
	25		>0.9999		0.9983	>0.9999	>0.9999	0.9163	0.9812				
	20		>0.9999		0.9587	0.8505	>0.9999						
	15		>0.9999				0.9494						
	10												
Peak II–III	Frequency (kHz)	4		8		12		16		24		32	
		4 weeks	7 weeks	4 weeks	7 weeks	4 weeks	7 weeks	4 weeks	7 weeks	4 weeks	7 weeks	4 weeks	7 weeks
Intensity (dB SPL)	80	0.8707	0.6113	0.689	>0.9999	0.9824	>0.9999	0.3588	0.978	0.372	0.5588	0.0045	0.1442
	75	0.9998	0.9919	0.9982	>0.9999	0.4251	>0.9999	0.1044	>0.9999	0.0181	0.4134	0.0179	0.0068
	70	0.9836	0.998	0.3577	>0.9999	0.238	>0.9999	0.1086	>0.9999	0.017	0.3246	0.0009	0.0165
	65	>0.9999	0.9876	0.4207	>0.9999	0.1467	>0.9999	0.3447	>0.9999	0.0066	0.149	0.0165	0.0528
	60	>0.9999	0.9487	0.1327	0.9931	>0.9999	>0.9999	0.4631	>0.9999	0.005	0.2074	0.4277	0.2029
	55	0.997	0.9829	0.0053	>0.9999	0.467	0.9982	0.4612	>0.9999	0.0598	>0.9999	0.2993	0.3881
	50	0.991	>0.9999	0.0375	>0.9999	0.3952	0.7112	0.3703	>0.9999	0.8698	0.92	0.0801	0.9978
	45	0.9994	>0.9999	0.1682	>0.9999	0.3314	0.703	0.6739	0.6934	0.181	0.9416	0.6623	>0.9999
	40	0.8853	0.9619	0.0487	0.9987	0.1598	>0.9999	0.9974	0.8068	0.0796	0.6959	0.0182	>0.9999
	35	0.9999	>0.9999	0.0497	0.9663	0.1338	>0.9999	0.1335	>0.9999		0.8289		
	30	>0.9999	>0.9999		>0.9999	0.0515	>0.9999	>0.9999	>0.9999				
	25		>0.9999		>0.9999	0.0699	0.9962	>0.9999	>0.9999				
	20		0.3171		>0.9999	>0.9999	>0.9999						
	15		>0.9999				0.9997						
	10		>0.9999										
Peak III–IV	Frequency (kHz)	4		8		12		16		24		32	
		4 weeks	7 weeks	4 weeks	7 weeks	4 weeks	7 weeks	4 weeks	7 weeks	4 weeks	7 weeks	4 weeks	7 weeks
Intensity (dB SPL)	80	0.4531	0.7043	0.205	0.4589	0.2502	0.9302	0.7793	0.9878	>0.9999	0.9931	0.9009	0.9988
	75	0.2013	0.8772	0.0368	0.0112	0.9956	0.9999	0.9855	0.9655	>0.9999	0.9856	0.9904	>0.9999
	70	0.1862	0.1238	0.0645	0.0138	>0.9999	0.8105	0.9989	0.0526	>0.9999	0.9992	>0.9999	0.9978
	65	0.1297	0.2098	0.1169	0.0385	0.9974	0.7555	0.9996	0.9163	0.9477	>0.9999	0.9987	0.9877
	60	0.0218	0.0046	0.759	0.3102	0.6116	0.4974	0.8846	0.9695	>0.9999	0.9987	>0.9999	0.8141
	55	0.0265	0.8982	0.9981	0.3534	0.6543	0.434	0.446	0.8257	>0.9999	0.8751	>0.9999	>0.9999
	50	0.0075	0.5608	>0.9999	0.1143	0.1651	0.9721	0.987	0.071	0.9922	>0.9999	0.9986	>0.9999
	45	0.1125	0.0212	>0.9999	0.3948	0.6763	0.8123	0.01	>0.9999	0.1943	>0.9999	0.9791	0.9304
	40	0.9884	0.8972	0.9933	0.4903	0.8028	>0.9999	0.2806	0.375	0.5351	>0.9999	0.8103	>0.9999
	35	0.9805	>0.9999	>0.9999	>0.9999	0.7441	0.5163	0.7894	0.9962		>0.9999		
	30		>0.9999		>0.9999	0.02	>0.9999	0.1785	>0.9999				
	25		0.9837		>0.9999	0.8812	>0.9999		>0.9999				
	20		>0.9999		0.9983		0.996						
	15		>0.9999				0.2176						
	10												
Peak I–III	Frequency (kHz)	4		8		12		16		24		32	
		4 weeks	7 weeks	4 weeks	7 weeks	4 weeks	7 weeks	4 weeks	7 weeks	4 weeks	7 weeks	4 weeks	7 weeks
Intensity (dB SPL)	80	0.9976	0.9757	0.9166	>0.9999	0.9616	>0.9999	0.554	0.5908	0.1734	>0.9999	<0.0001	0.992
	75	0.9889	>0.9999	>0.9999	>0.9999	0.4404	0.9985	0.2906	>0.9999	0.6515	>0.9999	0.004	0.5808
	70	0.9949	0.9968	0.9626	>0.9999	0.0359	>0.9999	0.1191	0.9978	0.8584	>0.9999	0.0076	0.9909
	65	0.9992	0.9974	0.982	>0.9999	0.5053	>0.9999	0.2001	>0.9999	0.9199	>0.9999	0.0157	0.976
	60	>0.9999	>0.9999	0.9933	>0.9999	0.9914	0.9987	0.166	>0.9999	0.6087	>0.9999	0.2889	0.7911
	55	>0.9999	>0.9999	0.9414	>0.9999	0.9852	0.9994	0.2362	0.9978	0.9968	>0.9999	0.5865	0.6075
	50	>0.9999	>0.9999	0.8017	>0.9999	0.8667	0.9892	0.644	>0.9999	0.9993	>0.9999	0.1887	0.9994
	45	0.7248	>0.9999	0.6885	>0.9999	0.779	0.9981	0.7463	0.9723	0.8109	0.8705	>0.9999	0.9997
	40	0.3971	>0.9999	0.223	>0.9999	0.2776	>0.9999	0.9953	0.986	0.5799	0.938	0.4215	0.9471
	35	0.914	>0.9999	0.0168	>0.9999	0.8794	>0.9999	0.1221	0.9956		0.9739		
	30	0.8953	>0.9999		>0.9999	0.6252	>0.9999	>0.9999	0.9994				
	25		>0.9999		>0.9999	0.2341	>0.9999	>0.9999	0.9996				
	20		0.8909		0.9996	0.8523	0.9995						
	15		>0.9999				>0.9999						
	10		>0.9999										
Peak I–IV	Frequency (kHz)	4		8		12		16		24		32	
		4 weeks	7 weeks	4 weeks	7 weeks	4 weeks	7 weeks	4 weeks	7 weeks	4 weeks	7 weeks	4 weeks	7 weeks
Intensity (dB SPL)	80	0.1527	>0.9999	0.0537	0.8727	0.0656	0.9993	0.0631	0.3408	0.2903	0.7543	0.0342	0.8997
	75	0.0381	>0.9999	0.2052	0.0598	0.1836	0.9992	0.0675	0.985	0.5212	0.8423	0.176	0.5412
	70	0.0453	0.9938	0.0292	0.2666	0.0581	0.9977	0.0406	0.1804	0.8919	0.9866	0.019	0.8785
	65	0.0512	0.9986	0.0632	0.4032	0.2361	>0.9999	0.0834	0.9933	>0.9999	0.9899	0.0121	0.7291
	60	0.0778	0.3014	0.4671	0.3574	0.269	>0.9999	0.0159	0.9976	0.7358	0.2971	0.7695	0.9994
	55	0.049	0.9989	>0.9999	0.5349	0.2612	0.894	0.0064	0.5279	0.9767	0.887	0.9608	0.7697
	50	0.012	0.9811	0.9702	0.2268	0.0231	0.9748	0.2159	0.4367	>0.9999	0.9998	0.8609	0.9998
	45	0.0023	0.5266	0.9705	0.9487	0.0931	0.9928	0.0021	0.874	>0.9999	0.9985	0.9866	0.8887
	40	0.0465	0.9852	0.1471	0.4967	0.0268	>0.9999	0.1306	0.3928	>0.9999	0.9871	>0.9999	0.844
	35	0.2528	>0.9999	0.0306	>0.9999	0.2056	>0.9999	0.0093	>0.9999		0.9265		
	30		>0.9999		>0.9999	0.0037	>0.9999	0.9117	0.9992				
	25		0.99		0.9985	0.0482	>0.9999		0.9969				
	20		0.8419		0.9084		0.9977						
	15		>0.9999				0.9148						
	10												
		Amplitudes (μV)											
Peak I	Frequency (kHz)	4		8		12		16		24		32	
		4 weeks	7 weeks	4 weeks	7 weeks	4 weeks	7 weeks	4 weeks	7 weeks	4 weeks	7 weeks	4 weeks	7 weeks
Intensity (dB SPL)	80	0.9999	>0.9999	0.5909	0.9978	0.2805	>0.9999	0.9878	0.9304	0.3953	0.9988	0.7775	>0.9999
	75	>0.9999	>0.9999	>0.9999	>0.9999	0.1747	>0.9999	0.7161	>0.9999	0.847	>0.9999	0.9956	>0.9999
	70	>0.9999	>0.9999	>0.9999	>0.9999	0.5717	0.9991	0.2999	>0.9999	0.9808	>0.9999	>0.9999	0.9994
	65	0.9999	0.9999	>0.9999	>0.9999	0.6683	>0.9999	0.4814	>0.9999	0.8283	0.9942	>0.9999	>0.9999
	60	>0.9999	0.9901	0.9999	0.9948	0.2169	>0.9999	0.6389	>0.9999	0.9728	0.999	0.9972	>0.9999
	55	>0.9999	>0.9999	>0.9999	0.9997	0.1693	>0.9999	0.7502	>0.9999	0.9996	0.9998	>0.9999	>0.9999
	50	0.9899	>0.9999	0.9989	0.8927	0.5948	>0.9999	0.9635	>0.9999	>0.9999	>0.9999	>0.9999	>0.9999
	45	0.407	>0.9999	0.9501	>0.9999	0.9006	>0.9999	0.9804	>0.9999	>0.9999	>0.9999	>0.9999	>0.9999
	40	0.3773	>0.9999	0.9979	>0.9999	0.8234	>0.9999	0.9985	>0.9999	>0.9999	>0.9999	>0.9999	>0.9999
	35	0.7805	>0.9999	>0.9999	>0.9999	0.9977	>0.9999	0.999	>0.9999		>0.9999		
	30	0.9858	>0.9999		>0.9999	0.9999	>0.9999	>0.9999	>0.9999				
	25		>0.9999		>0.9999	>0.9999	>0.9999	>0.9999	>0.9999				
	20		>0.9999		>0.9999	>0.9999	0.9984		>0.9999				
	15		>0.9999				>0.9999						
	10												
Peak II	Frequency (kHz)	4		8		12		16		24		32	
		4 weeks	7 weeks	4 weeks	7 weeks	4 weeks	7 weeks	4 weeks	7 weeks	4 weeks	7 weeks	4 weeks	7 weeks
Intensity (dB SPL)	80	0.0041	>0.9999	>0.9999	>0.9999	>0.9999	>0.9999	0.9987	0.995	0.0536	0.0714	0.6777	0.1534
	75	0.967	0.9933	0.2544	0.9966	0.7625	>0.9999	0.5765	0.799	0.0293	0.4901	0.6882	0.9988
	70	>0.9999	0.9162	0.0546	0.8915	0.0544	>0.9999	0.1351	0.9213	0.8061	0.982	0.986	0.9787
	65	>0.9999	0.921	0.011	0.916	0.0114	0.9853	0.263	0.7202	>0.9999	>0.9999	0.9997	0.9996
	60	0.9997	0.9992	0.0216	0.94	0.0006	0.9953	0.3549	0.9947	0.9995	>0.9999	0.9972	>0.9999
	55	>0.9999	>0.9999	0.0685	0.9992	<0.0001	0.8365	0.1353	>0.9999	>0.9999	>0.9999	>0.9999	>0.9999
	50	0.9983	>0.9999	0.1122	0.9952	0.0002	0.987	0.1224	>0.9999	>0.9999	>0.9999	>0.9999	>0.9999
	45	0.7312	>0.9999	0.0227	>0.9999	0.0002	0.999	0.1396	>0.9999	>0.9999	0.9996	>0.9999	0.9673
	40	0.4724	0.9999	0.1345	>0.9999	0.0001	>0.9999	0.3741	0.9997	0.91	0.9753	>0.9999	0.998
	35	0.6146	>0.9999	0.713	>0.9999	0.0131	>0.9999	0.4482	0.9855		0.9949		
	30	0.9654	0.9977		>0.9999	0.2393	>0.9999	0.8649	>0.9999				
	25		>0.9999		>0.9999	0.86	>0.9999	>0.9999	>0.9999				
	20		>0.9999		>0.9999	0.9986	>0.9999						
	15		>0.9999				>0.9999						
	10												
Peak III	Frequency (kHz)	4		8		12		16		24		32	
		4 weeks	7 weeks	4 weeks	7 weeks	4 weeks	7 weeks	4 weeks	7 weeks	4 weeks	7 weeks	4 weeks	7 weeks
Intensity (dB SPL)	80	0.8773	0.9694	0.617	0.8557	0.9879	0.9979	>0.9999	>0.9999	0.9991	>0.9999	0.7727	0.9997
	75	0.0467	0.9852	0.4543	0.9824	0.9999	0.9997	0.198	>0.9999	>0.9999	0.9937	>0.9999	>0.9999
	70	0.0219	>0.9999	0.25	>0.9999	0.0625	>0.9999	0.0036	>0.9999	>0.9999	0.9996	>0.9999	0.6554
	65	0.021	>0.9999	0.0496	0.9989	0.0004	>0.9999	0.0003	0.8926	>0.9999	>0.9999	0.971	0.9484
	60	0.1528	0.809	0.0245	0.9994	<0.0001	0.8908	0.0017	0.9971	0.9999	>0.9999	>0.9999	0.9655
	55	0.8908	0.9668	0.2514	0.9982	0.0001	0.752	0.0503	>0.9999	0.9949	0.9047	>0.9999	0.982
	50	0.3084	>0.9999	0.718	0.9745	0.0039	0.9942	0.753	0.9988	0.9157	>0.9999	>0.9999	>0.9999
	45	0.5522	0.9984	0.8589	0.8129	0.2383	0.9858	>0.9999	0.9992	>0.9999	>0.9999	0.9161	0.882
	40	0.5642	>0.9999	0.9969	>0.9999	0.9678	>0.9999	>0.9999	0.9896	0.9999	0.9408	0.9908	>0.9999
	35	0.8501	0.9999	0.9998	0.9994	0.9973	0.9991	>0.9999	0.9882		>0.9999		
	30	0.9992	0.9996		0.9914	>0.9999	0.9423	>0.9999	>0.9999				
	25		0.9874		0.9937	>0.9999	>0.9999	>0.9999	>0.9999				
	20		>0.9999		>0.9999	>0.9999	>0.9999						
	15		>0.9999				>0.9999						
	10												
Peak IV	Frequency (kHz)	4	8		12		16		24		32	
		4 weeks	7 weeks	4 weeks	7 weeks	4 weeks	7 weeks	4 weeks	7 weeks	4 weeks	7 weeks	4 weeks	7 weeks
Intensity (dB SPL)	80	>0.9999	0.9937	>0.9999	>0.9999	0.8751	>0.9999	>0.9999	0.9994	0.0971	>0.9999	0.2365	0.8811
	75	0.9989	0.9997	>0.9999	0.9999	>0.9999	>0.9999	0.3295	0.9867	0.2672	>0.9999	0.874	>0.9999
	70	0.2896	0.9992	0.6309	0.9988	0.4904	0.9969	0.0242	0.9103	0.078	0.989	>0.9999	0.9995
	65	0.0589	>0.9999	0.0544	>0.9999	0.2406	>0.9999	0.0022	0.9991	0.2641	>0.9999	0.9889	0.9989
	60	0.4861	>0.9999	0.0043	>0.9999	0.0091	>0.9999	0.0083	0.9603	0.5788	>0.9999	0.9982	0.8113
	55	0.6744	>0.9999	0.1152	>0.9999	0.0302	0.9997	0.008	>0.9999	0.9929	>0.9999	0.7973	>0.9999
	50	0.8413	>0.9999	0.7501	0.8958	0.0483	0.9999	0.0039	0.9123	0.9871	0.9959	0.9664	0.9934
	45	0.5569	>0.9999	0.1376	>0.9999	0.028	0.9688	0.2175	>0.9999	0.8922	0.9975	0.9352	>0.9999
	40	0.4028	0.9885	0.7979	0.9996	0.2109	>0.9999	0.2769	>0.9999	0.3515	>0.9999	>0.9999	0.7964
	35	0.9667	>0.9999	0.9995	>0.9999	0.4729	>0.9999	0.8783	>0.9999		>0.9999		
	30		0.9994		>0.9999	0.9922	0.9997	>0.9999	0.9866				
	25		0.9995		>0.9999	0.7008	>0.9999		>0.9999				
	20		>0.9999		>0.9999		>0.9999						
	15		>0.9999				>0.9999						
	10												
													

### Experimental design and statistical analysis

We used multiple litters for each experimental group. Each litter contained pups injected with DMSO or BLZ945 to account for the variability between litters. Quantitative results are presented as mean scores ± SEM. Statistical analysis was done using Prism Software (v8.4.1; GraphPad Software). Comparisons between different treatment (control and BLZ945) and age groups (three and four weeks or four and seven weeks) were made using two-way ANOVA with Sidak’s multiple comparisons test unless otherwise indicated. Statistical significance was accepted at *p *<* *0.05. Details of statistical analyses are presented in Tables 3-5.

## Results

### Microglia repopulate the MNTB after cessation of treatment

Treatment with BLZ945 early in development (repeated subcutaneous injections at P2, P4, P6, P8, and P10) was used to eliminate microglia in the brainstem of mice as previously described (Milinkeviciute et al., 2019). We first determined whether microglia return after treatment cessation and if so, to what extent and over what time course. We perfused mice at several posttreatment time points: P14, P18, three, four, and seven weeks (Fig. 1*A*). We used four DMSO and 4 BLZ945-injected mice at P14, three DMSO and 4 BLZ945-treated mice at P18, five DMSO and five BLZ945-treated mice at three weeks, six DMSO and six BLZ945-treated mice at four weeks and four DMSO and seven BLZ945-injected mice at seven weeks. Littermate controls injected with DMSO were used in each age group. Each cohort consisted of animals from a few different litters to account for the variability between litters. Microglia were identified using IBA1 immunofluorescence. The areal coverage of IBA1 immunolabel was used to evaluate the extent of microglia repopulation at four and seven weeks (Fig. 1*B*). There was no significant difference in the IBA1 areal coverage in the MNTB (four-week DMSO: 0.06 ± 0.004; four-week BLZ945: 0.05 ± 0.01; seven-week DMSO: 0.04 ± 0.01; seven-week BLZ945: 0.04 ± 0.003) between control and drug treated animals at both four (*p *=* *0.0574, *t *=* *2.360; df = 19) and seven (*p *=* *0.9113, *t *= 0.3882; df = 19) weeks. IBA1 immunolabeling significantly decreased with age only in control animals (DMSO: *p *= 0.0166, *t *=* *2.943; df = 19; BLZ945: *p *=* *0.8425, *t *=* *0.5286; df = 19), but there was no difference between DMSO controls and BLZ945-treated mice at seven weeks (*p *=* *0.9113, *t *=* *0.3882; df = 19; Fig. 1*B*).

Microglia densely populated VCN, LSO, and MNTB of control mice in each age group (Fig. 1*C–G*). In BLZ945-injected mice, microglia were not observed in the VCN, LSO or MNTB at P14 (Fig. 1*H*). A few microglial cells were seen in the VCN at P18 but no IBA1 staining was observed in the LSO or MNTB (Fig. 1*I*). By three weeks, microglia were seen throughout VCN and in LSO, but not in MNTB (Fig. 1*J*). At four and seven weeks, microglia were present in all three auditory brainstem nuclei (Fig. 1*K*,*L*). This gradual microglial repopulation, first in VCN, then LSO, then MNTB reflects an overall lateral-to-medial movement of microglial emergence that is seen throughout the brainstem in our sections, reflecting a similar trend to how microglia occupy the brainstem during the first two postnatal weeks (Dinh et al., 2014). Thus, microglial repopulation of MNTB takes place between the ages of three and four weeks, or 11–18 d after the treatment is discontinued.

### GFAP expression in the MNTB returns to control levels after return of microglia

GFAP is regarded as a marker of mature (Wofchuk and Rodnight, 1995; Gomes et al., 1999; Middeldorp and Hol, 2011) or active astrocytes (Eng and Ghirnikar, 1994; Fuentes-Santamaria et al., 2017). In MNTB, GFAP expression changes during development, with no expression at P0, very sparse expression at P6, and increased density at P14. GFAP staining in VCN is first observed at P14 and some positive fibers are seen in MNTB at P23 (Dinh et al., 2014). We looked at 20× confocal images of sections through the VCN, LSO, and MNTB stained for IBA1 and GFAP at three weeks (11 d postcessation of treatment) in three DMSO and three BLZ945-treated mice. As noted above, microglia repopulated auditory brainstem sequentially, starting from the most peripheral nuclei. At three weeks, microglia were found throughout the VCN, LSO, and MNTB of control mice. In VCN, GFAP labeling was mostly confined to outer layer of the nucleus with sparse labeling in the deeper layers. In LSO, GFAP-positive cells were also sparse while MNTB appeared to be the most populated (Fig. 2*A*). Similar to control animals, in BLZ945-injected animals, microglia were spread throughout the VCN, however, in the LSO fewer cells were observed, and they appeared larger than in the VCN or LSO of control animals. MNTB was still devoid of IBA1-positive cells. In VCN GFAP was largely restricted to outer layers. GFAP staining in LSO was also very similar to that of control mice. In MNTB, GFAP-positive cells were observed throughout the nucleus (Fig. 2*B*). We showed that microglia fully repopulated MNTB by the age of four weeks, thus, next we focused on GFAP expression at four and seven weeks.

When microglia were depleted early postnatally, GFAP areal coverage was significantly reduced at P13 (Milinkeviciute et al., 2019). We examined GFAP areal immunofluorescence coverage ratio in 11 control and six BLZ945-treated mice at four weeks as well as six control and eight BLZ945-injected animals at seven weeks. Abundant GFAP was observed in four-week-old control mice (four-week DMSO: 0.05 ± 0.01; Fig. 2*C*). In drug treated four-week animals GFAP expression (four-week BLZ945: 0.04 ± 0.01; Fig. 2*D*) was localized more to the peripheral parts of the MNTB as reported in Milinkeviciute et al. (2019) at P13 while in control tissue GFAP labeling was found throughout the MNTB (Fig. 2*A*). At seven weeks, both, control and BLZ945-treated mice showed abundant GFAP labeling throughout the MNTB (seven-week DMSO: 0.04 ± 0.003; seven-week BLZ945: 0.04 ± 0.002 (Fig. 2*E–G*). The areal GFAP coverage was significantly reduced in BLZ945 injected mice at four weeks (*p *=* *0.0258, *t *=* *2.66; df = 27) but was comparable to control levels at seven weeks (*p *=* *0.8450, *t *=* *0.5215; df = 27; Fig. 2*G*). There was no significant age-related change in GFAP areal coverage ratio in any of the two experimental groups (DMSO: *p *=* *0.2535, *t *=* *1.537; df = 27; BLZ945: *p *=* *0.8381, *t *=* *0.5341; df = 27). These results suggest a relationship between microglia and the GFAP-positive astrocytic population in MNTB.

### MNTB neurons are monoinnervated after microglia recovery

**Figure 3.**
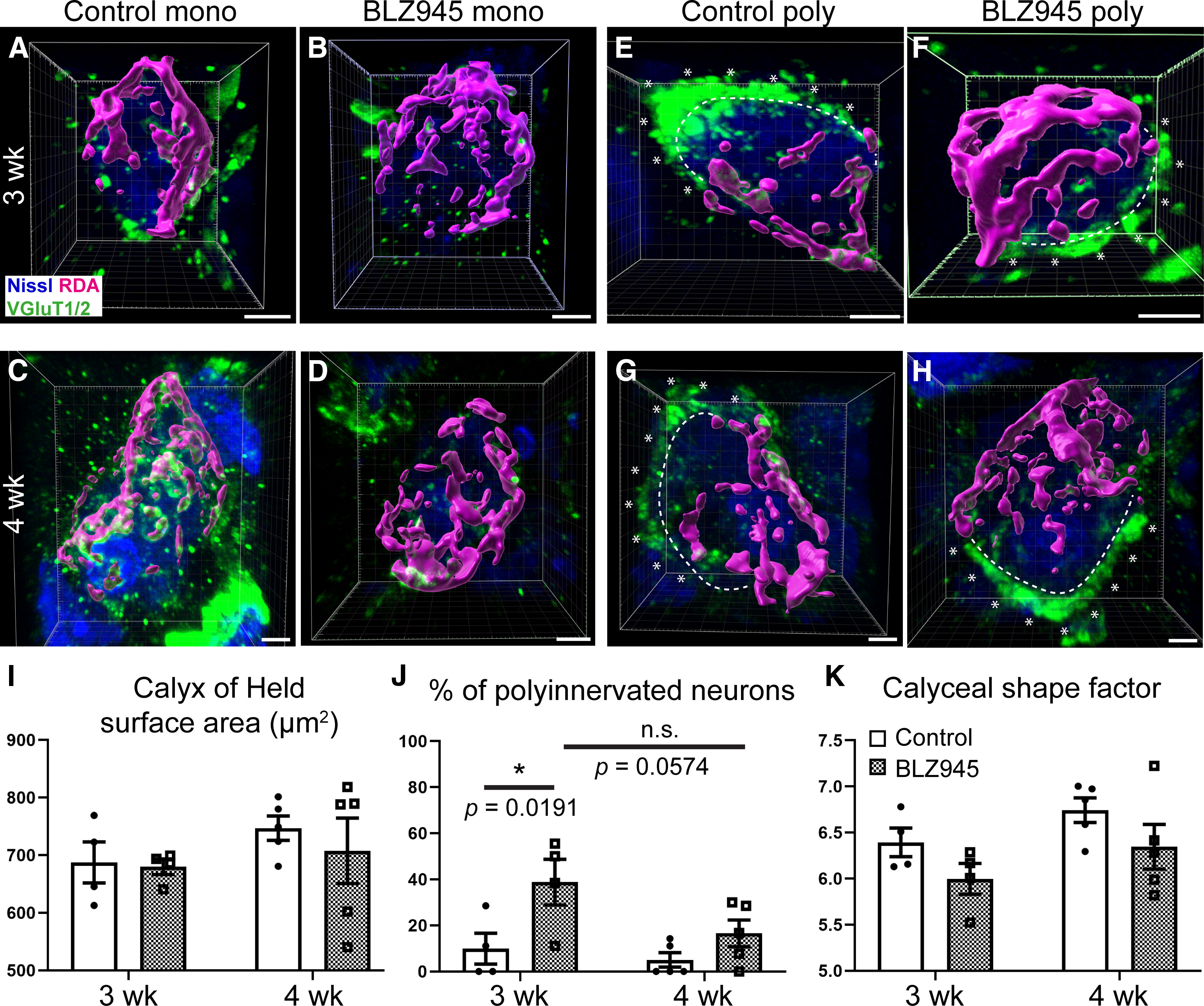
Confocal analysis of MNTB neurons. ***A***, Monoinnervated neuron from a three-week-old control mouse. Reconstructed RDA-labeled calyx of Held (magenta); MNTB neuron (blue); and VGLUT1/2 labeling (green). ***B***, Monoinnervated neuron from a three-week-old BLZ945-treated mouse. ***C***, ***D***, Monoinnervated neuron from a four-week-old control and BLZ945-injected mouse, respectively. ***E***, ***F***, Polyinnervated neuron from a three-week-old control and BLZ945-injected mouse, respectively. Dashed line indicates the contour of the neuron while asterisks show the VGLUT1/2-positive terminal contacting the same neuron. ***G***, ***H***, Polyinnervated neuron from a four-week-old control and BLZ945-treated animal, respectively. An extra calyceal input was determined by a continuous VGLUT1/2 staining outside of the RDA-positive area (asterisks) surrounding the MNTB neuron. ***I***, Comparison of calyx of Held surface area in control and BLZ945-treated mice at three and four weeks. There was no difference between the two animal groups at both ages tested. No age-related increase in calyceal area was observed. ***J***, Comparison of polyinnervated MNTB neurons as a percentage of MNTB neurons analyzed revealed significantly more polyinnervated neurons in BLZ945-injected mice at three weeks when compared with controls. No difference between control and BLZ945 injected mice was found at four weeks. The age-related change in BLZ945-injected animals did not reach significance (*p *=* *0.0574). Scale bars: 5 μm (***A***, ***B***, ***E***, ***F***), 3 μm (***C***, ***G***, ***H***), and 4 μm (***D***).

We labeled calyces of Held in the MNTB on both sides by injecting RDA into the VAS at the midline. Tissue was labeled with VGLUT1/2 antibody to identify excitatory inputs, which in MNTB mainly corresponds to calyces of Held (Billups, 2005). MNTB neurons contacted by an RDA labeled calyx were used for confocal imaging and subsequent reconstruction in 3D as previously described in Milinkeviciute et al. (2019). We reconstructed and analyzed calyces of Held from four DMSO and four BLZ945 at three weeks and five DMSO and five BLZ945-injected mice at four weeks. Monoinnvervated and polyinnervated MNTB neurons were identified in both control and BLZ945-treated mice at three weeks (Fig. 3*A–D*) and four weeks (Fig. 3*E–H*). We evaluated each MNTB neuron for VGLUT1/2 labeled inputs outside the RDA-labeled calyx (see Materials and Methods) to determine the percentage of neurons contacted by more than one calyx (three-week DMSO: 9.92 ± 6.75; three-week BLZ945: 38.78 ± 9.89; four-week DMSO: 5.08 ± 3.15; four-week BLZ945: 16.59 ± 5.82). At three weeks, the percentage of polyinnervated neurons was significantly increased in the BLZ945-treated mouse group compared with their age-matched controls (*p *=* *0.0191, *t *=* *2.998; df = 14). We found no significant difference in polyinnervation of MNTB neurons between control and drug treated animals at four weeks (*p *=* *0.3645, *t *=* *1.336; df = 14; Fig. 3*I*) Numbers of animals and calyces analyzed are presented in Table 2. These results suggest that neurons in MNTB remain polyinnervated when microglia are absent from MNTB.

We measured the surface area and volume of RDA-positive and reconstructed calyces from monoinnervated neurons. Calyces in the MNTB of control and drug injected mice did not differ in size at three weeks (*p *=* *0.9895, *t *=* *0.131; df = 14) or four weeks (*p *=* *0.7005, *t *=* *0.7724; df = 14). There was no significant age-related change in calyx surface area in control or BLZ945-treated mice (DMSO: *p *=* *0.4941, *t *=* *1.103; df = 14; BLZ945: *p *=* *0.8527, *t *=* *0.5126; df = 14; Fig. 3*J*). Complexity of calyces was measured by calculating a shape factor using the formula described in see Materials and Methods (Bogaerts et al., 2009; Muniak et al., 2018). Calyces did not become more complex with age (DMSO: *p *=* *0.3715, *t *=* *1.323; df = 14; BLZ945: *p *=* *0.3717, *t *=* *1.322; df = 14; Fig. 3*K*). There was no difference observed between the intricacy of calyceal branching between control and BLZ945-injected mice at three weeks (*p *=* *0.3210, *t *= 1.425; df = 14) or at four weeks (*p *=* *0.2488, *t *=* *1.594; df = 14; Fig. 3*K*; Table 3). In summary, temporary microglial depletion and subsequent repopulation does not seem to affect the growth and morphologic maturation (in terms of branching complexity) of calyces of Held.

### Temporary postnatal microglia elimination alters ABRs

#### Temporary microglia depletion results in elevated hearing thresholds

**Figure 4.**
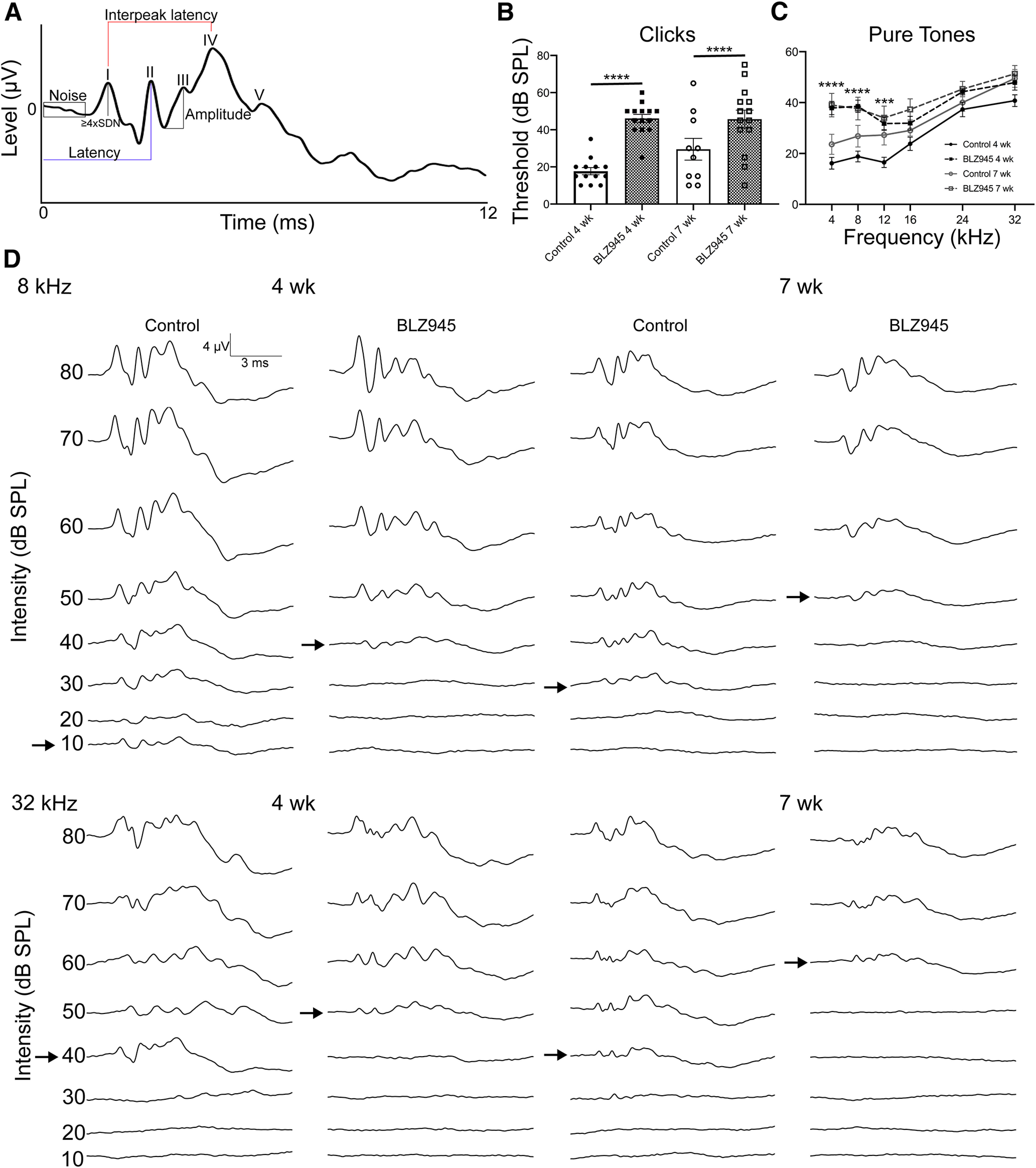
Effect of temporary BLZ945 treatment on auditory thresholds. ***A***, Example trace for method of ABR analysis. Threshold was determined as the sound intensity for which peak I amplitude was greater than or equal to SDs above the averaged noise before the beginning of the ABR trace. Latency (blue) is the time of the apex of the peak from stimulus onset. Interpeak latency (red) is the difference in time between two respective peaks. Amplitude (black) is the difference in microvolts between the preceding trough to the apex of the peak. ***B***, Averaged thresholds for responses to click stimuli at four and seven weeks. BLZ945 injected mice had significantly elevated thresholds compared with their age-matched controls at four and seven weeks. ***C***, Averaged auditory thresholds for responses to pure tone stimuli at four weeks (black) and seven weeks (gray). BLZ945-treated animals had significantly elevated thresholds in response to 4, 8, and 12 kHz at four weeks. Treatment did not affect thresholds at seven weeks, compared with age-matched controls. ***D***, Example traces in response to pure tones in control and BLZ945-treated mice at four and seven weeks of age. Thresholds are indicated with black arrows.

Auditory brainstem responses were measured in mice at four weeks (*n* = 13 DMSO, *n* = 14 BLZ945) and seven weeks (*n* = 11 DMSO, *n* = 15 BLZ945). Click and pure tone stimuli were presented to the left ear at decreasing intensities from 80 dB SPL to 10 dB SPL. Click and pure tone traces were analyzed for threshold, the lowest intensity at which peak I level (μV) was equal to or exceeded the 4 SDs above the mean noise level (Fig. 4*A*). BLZ945-treated mice had significantly higher thresholds in response to clicks at both four and seven weeks (four weeks: *p < *0.0001, df = 47; seven weeks *p = *0.0321, df = 47; one-way ANOVA with Tukey’s multiple comparisons; Fig. 4*B*). Age did not affect thresholds in both treatment groups (control: *p = *0.1884, df = 47; BLZ945: *p = *0.9999, df = 47; one-way ANOVA with Tukey’s multiple comparisons; Fig. 4*B*). At four weeks, BLZ945-treated mice displayed elevated thresholds in response to pure tones at 4, 8, and 12 kHz (4 kHz: *p *<* *0.0001, df = 24.89; 8 kHz: *p *<* *0.0001, df = 24.80; 12 kHz: *p *=* *0.0007, df = 23.65; 16 kHz: *p *=* *0.2228, df = 23.98; 24 kHz: *p *=* *0.2179, df = 16.58; 32 kHz: *p *=* *0.3584, df = 24.08; Fig. 4*B*). At seven weeks, BLZ945-treated mice displayed pure tone thresholds that were comparable to control, and there were no significant differences at any frequency tested (4 kHz: *p* = 0.1043, df = 24.0; 8 kHz: *p* = 0.4413, df = 23.63; 12 kHz: *p* = 0.8559, df = 24.0; 16 kHz: *p* = 0.4524, df = 20.73; 24 kHz: *p* = 0.8303, df = 20.54; 32 kHz: *p* = 0.9994, df = 22.56; Fig. 4*C*). Example traces indicating thresholds from each group at low and high frequencies are displayed in Figure 4*D*. These results indicate a temporary hearing impairment as a result of CSF1R inhibition during development and recovery after a prolonged period of microglial repopulation.

#### Peak latencies are increased in BLZ945-treated animals

Peaks in the ABR waveform reflect neuronal activity in different parts of the auditory pathway. Peak I indicates activity in the cochlea, spiral ganglion neurons (SGN), and VIIIth nerve, peak II represents, the cochlear nucleus (CN), peak III, the superior olivary complex (SOC), and peak IV, the lateral lemniscus (LL; Jewett et al., 1970; Jewett and Williston, 1971; Picton et al., 1974; Henry, 1979). Mice were tested at four and seven weeks to compare the auditory function at early and late time points after microglial repopulation. All ABR statistical analyses including input-output simple linear regressions are displayed in Tables 4, 5.

**Figure 5.**
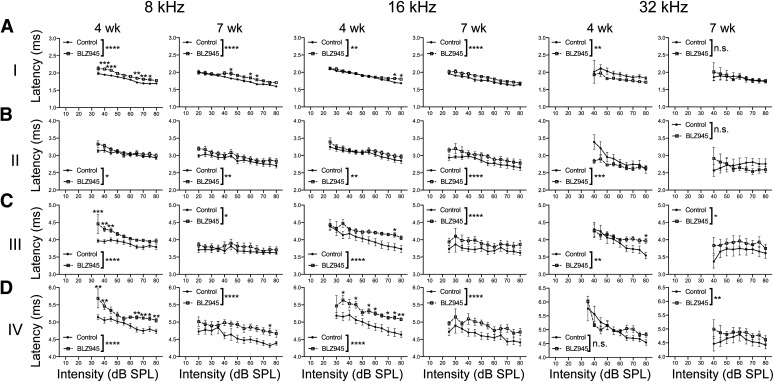
Effect of temporary BLZ945 treatment on ABR peak latencies. ***A***, Absolute peak I latency in response to 8-, 16-, and 32-kHz stimuli at four and seven weeks. Treatment with BLZ945 early in development resulted in significantly increased latency at the low and middle frequencies but significantly decreased latencies at the higher frequencies at four weeks. By seven weeks, the latencies at the lower frequencies remained elevated but latencies at the high frequencies were comparable to control. ***B***, Peak II latency was significantly increased in the BLZ945-treated group. Latencies remained elevated by seven weeks at all frequencies except 32 kHz. ***C***, BLZ945 treatment resulted in significantly increased peak III latency, with evidence of some recovery by seven weeks in all frequencies. ***D***, Significantly increased latencies in peak IV were detected in BLZ945-treated mice in low and middle frequencies at four weeks. Some improvement was detected by seven weeks, but 32-kHz stimuli resulted in significantly longer latency in the BLZ945 group.

Peak latency was determined as the time (ms) from stimulus at which the apex of the peak was detected (Fig. 4*A*). At four weeks, BLZ945-treated mice showed significantly increased peak I latencies in lower and middle frequencies. Higher frequencies displayed significantly decreased peak I latencies (Fig. 5*A*). At seven weeks, treatment effects on peak I latencies were still observed at the lower and middle frequencies, but no differences were seen at the higher frequencies (Fig. 5*A*), indicating some recovery in peak I latencies. Peak II latency in BLZ945-treated mice was significantly increased in low and middle frequencies but significantly decreased in high frequencies at four weeks (Fig. 5*B*). By seven weeks, peak II latency remained significantly delayed in the lower and middle frequencies but recovered at high frequencies (Fig. 5*B*). BLZ945 treatment led to significantly increased peak III latency (Fig. 5*C*) in most frequencies tested at four weeks. These treatment effects remained at some frequencies at seven weeks, but multiple comparison tests did not show any intensity-specific differences (Tables 4, 5), suggesting an overall decrease in the effect of the treatment. In peak IV (Fig. 5*D*), BLZ945 treatment resulted in significantly increased latencies at four weeks in the lower and middle frequencies, but not the high frequencies. At seven weeks, latencies showed some recovery in the middle frequencies, but BLZ945-treated mice still showed significantly increased latencies at 32 kHz (Fig. 5*D*). Together, these results indicate that temporary CSF1R inhibition early in development affects peak latencies at four weeks. Some, but not all, of these latency changes recover after cessation of drug treatment by seven weeks.

#### Increased interpeak latencies partially recover by seven weeks of age

**Figure 6.**
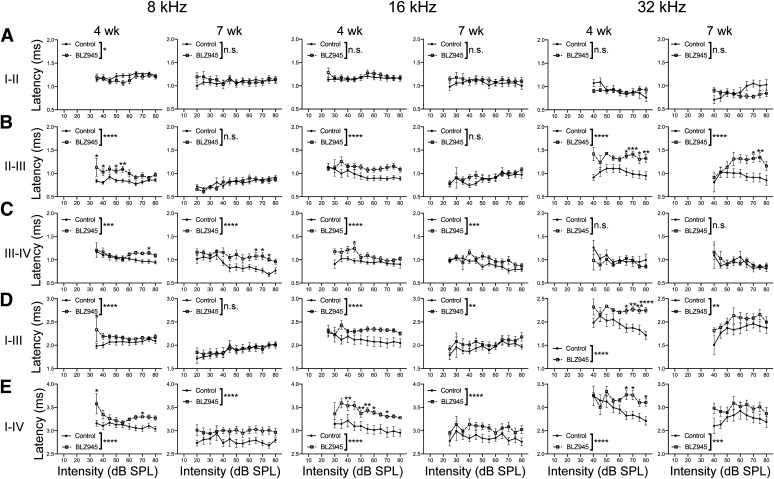
Effect of temporary BLZ945 treatment on ABR interpeak latencies. ***A***, ABR peak I–II interpeak latencies in response to 8 kHz were significantly decreased in BLZ945-treated mice at four weeks. By seven weeks, there were no differences at any frequency level. ***B***, Peak II–III interpeak latency was significantly increased in BLZ945-treated mice at all frequency levels. By seven weeks, low and middle frequencies recovered, but high frequencies remained significantly elevated. ***C***, Peak III–IV interpeak latencies for the BLZ945 group were significantly longer in low and middle frequencies in both ages, while the high frequencies were not affected. ***D***, Peak I–III interpeak latency was significantly elevated in BLZ945-treated mice at all frequency levels at four weeks. By seven weeks, only the low frequency was comparable to the control group. ***E***, BLZ945 treatment resulted in significantly elevated peak I–IV interpeak latency at all frequencies in both ages.

We next measured interpeak latencies to test whether the observed latency differences in the BLZ945-treated mice is because of peripheral conduction defects, reflect any delays in central activity or is a result of both. Interpeak latency was determined as the difference in time between the apex of the peak and the apex of the preceding peak (ms). At four weeks, treatment effects on interpeak latencies were observed between peaks I and II at 8 and 24 kHz, where BLZ945-treated mice showed significantly decreased interpeak latencies. However, by seven weeks, there were no differences in peak I–II interpeak latencies, indicating that cochlea-SGN-VIIIth nerve signal transduction recovered (Fig. 6*A*). BLZ945 treatment led to significantly increased peak II–III interpeak latencies at four weeks at 8, 12, 16, 24, and 32 kHz (Fig. 6*B*). At seven weeks, BLZ945-treated mice showed normal peak II–III latencies at the lower and middle frequencies, but the higher frequencies remained delayed (Fig. 6*B*). Together, these data suggest that signal transmission from the CN to the SOC is delayed after temporary microglia depletion but recovers by at seven weeks. Peak III–IV interpeak latencies (Fig. 6*C*) were significantly elevated at 4, 8, 12, 16, and 24 kHz in BLZ945-treated mice at four weeks. *Post hoc* multiple comparison analyses showed that these interpeak latency differences were more apparent in the lower frequencies. Similar results were obtained at seven weeks, indicating that these increases in peak III–IV latencies do not recover with time (Fig. 6*C*).

To investigate the overall effects of temporary CSF1R inhibition on central latencies, we measured interpeak latencies between peaks I–III and I–IV. BLZ945 treatment led to increased interpeak latencies between peaks I and III at four weeks at all frequencies tested when compared with control mice. At seven weeks, peak I–III latencies improved at most frequency levels except for 16 and 32 kHz (Tables 4, 5). Treatment effect comparisons for peak I–IV latencies showed a significant increase in the BLZ945-treated group at all frequencies tested at both four and seven weeks compared with controls (Fig. 6*D*). However, *post hoc* comparisons did not display any significant changes for any particular intensity at seven weeks, indicating some recovery in central latencies after prolonged microglia repopulation. Together, these findings demonstrate that BLZ945 treatment in the early postnatal period increases latencies in the auditory brainstem measured at four weeks, and that these effects are partially improved by seven weeks.

#### Peak amplitudes are diminished at four weeks but recover by seven weeks of age

**Figure 7.**
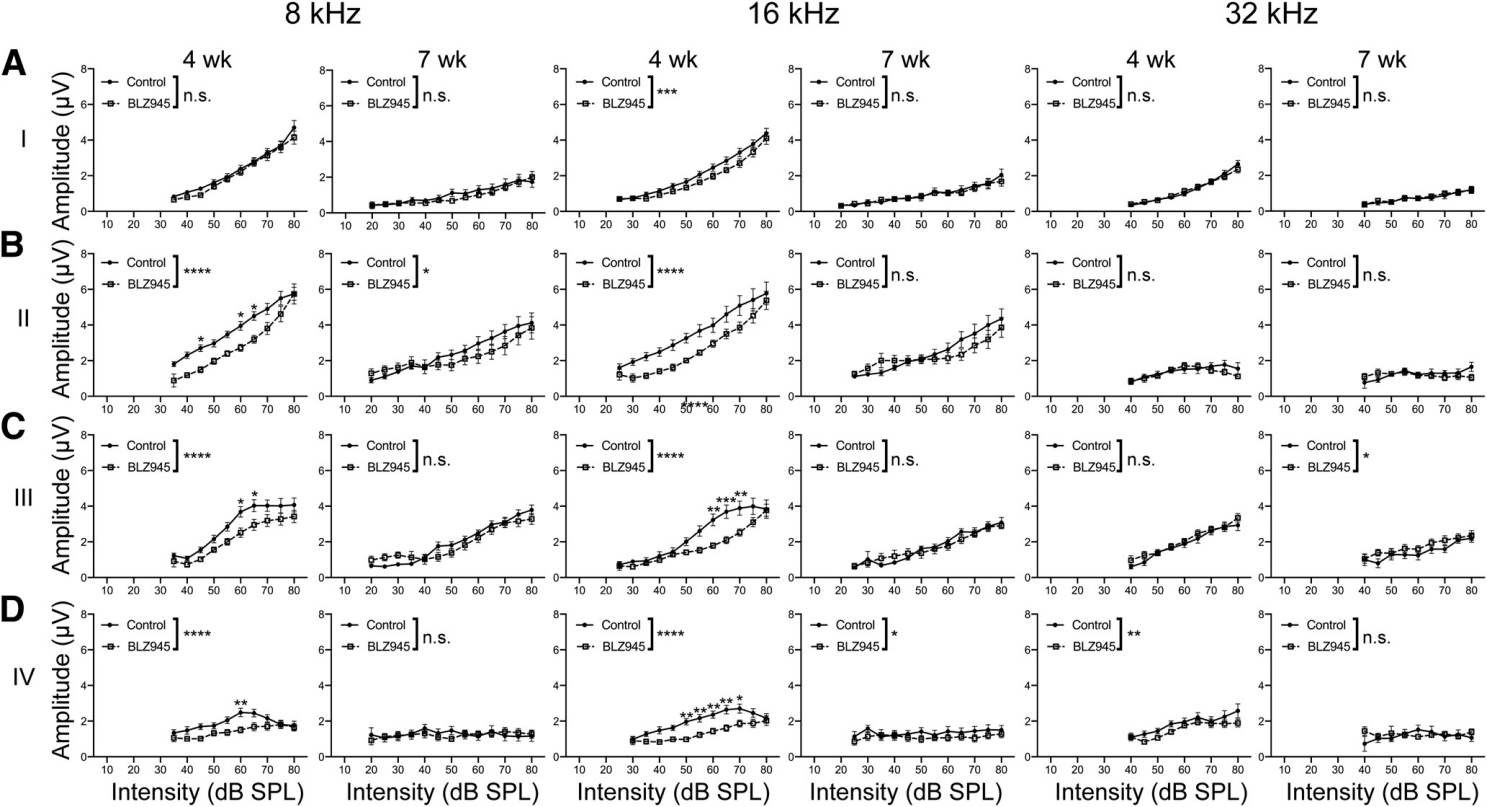
Effect of temporary BLZ945 treatment on ABR amplitudes. ***A***, Trough to peak amplitudes (μV) of ABR peak I in response to 8-, 16-, and 32-kHz stimuli at four and seven weeks. Amplitudes were comparable to control at most frequency levels except 16 kHz, where amplitudes were significantly diminished. ***B***, Peak II amplitude was significantly decreased in the BLZ945-treated group at the low and middle frequencies, with some improvement by seven weeks. ***C***, Peak III amplitudes were significantly lower in the BLZ945 mice at low and middle frequencies at four weeks, and recovered by seven weeks. The higher frequency showed that peak III amplitude was significantly elevated in the BLZ945-treated group at seven weeks. ***D***, BLZ945-treated mice showed significantly decreased peak IV amplitudes at all frequency levels at four weeks. By seven weeks, peak IV amplitude was comparable to control at 8 and 32 kHz.

We next examined the effect of BLZ945 treatment on ABR peak amplitudes, determined as the difference in μV from the preceding trough to the following peak. Peak I (Fig. 7*A*) amplitude showed that there was a significant decrease in treated animals across all frequencies except 8 and 32 kHz at four weeks. *Post hoc* multiple comparisons tests did not show significant differences at any specific intensity at all frequency levels. At seven weeks, peak I amplitude did not differ between BLZ945-treated mice and control mice. Peak II amplitude (Fig. 7*B*) was significantly diminished at 8, 12, 16, and 24 kHz at most intensities in BLZ945-treated mice at four weeks. By seven weeks, peak II amplitudes were comparable to control levels at all frequencies and intensities (Fig. 7*B*). BLZ945 treatment at four weeks resulted in diminished peak III amplitudes at the low and middle frequency levels (4, 8, 12, and 16 kHz; Fig. 7*C*). By seven weeks, peak III amplitudes in treated animals were comparable to controls at all frequencies and intensities, except for 32 kHz where a significant increase in amplitude was detected in the BLZ945-treated group (Fig. 7*C*). Peak IV (Fig. 7*D*) amplitudes were significantly decreased in BLZ945-trreated animals at four weeks at all frequency levels. By seven weeks, amplitudes at most frequencies recovered, except for 16 kHz where a significant decrease in amplitude was detected in BLZ945-treated mice (Fig. 7*D*). Multiple comparison analyses revealed no difference at any intensity at seven weeks, indicating that peak IV amplitudes were generally recovered. Overall, these data show that at four weeks, peak amplitudes are significantly decreased in BLZ945-treated animals compared with controls (see Tables 4, 5). After prolonged microglial repopulation, there is evidence of recovery of peak amplitudes throughout the auditory brainstem pathways.

## Discussion

In the present study, we used a CSF1R inhibitor to eliminate microglia early in development and then withdrew this drug to study microglia repopulation and its effects on auditory brainstem circuits. Previously, it was shown that such microglial depletion led to a decrease in GFAP expression as well as retention of polyinnervation in MNTB after the onset of hearing (Milinkeviciute et al., 2019). In this study, we showed that microglia recolonized the brainstem over an extended time course and polyinnervation was present when microglia were still absent from the MNTB at three weeks of age. The majority of MNTB neurons were no longer polyinnervated in BLZ945-treated mice at four weeks of age, a time when microglial numbers were comparable to control levels. Reduced GFAP expression took longer to reach that of controls; however, it was rectified by seven weeks. Functionally, early microglia depletion led to defects in auditory peak latencies and amplitudes, which largely recovered by the later age. This study emphasizes the plasticity of the brain after a challenge to the system and calls attention to the relationship between microglia and auditory function and between different glial cell types.

### Microglial repopulation

Microglia can be eliminated using genetic methods (Bruttger et al., 2015; Wieghofer et al., 2015), pharmacological methods (Pyonteck et al., 2013; Gerber et al., 2018), or irradiation (Simard and Rivest, 2004; Han et al., 2019). We used subcutaneous BLZ945 injections early in development and achieved a complete elimination of IBA1-positive cells in the brainstem. Oral BLZ945 treatment in the five-week cuprizone mouse model resulted in reduced microglial numbers (up to 60%; Beckmann et al., 2018), highlighting the differences between the delivery method as well as age of the animal and region of the brain analyzed despite of the same inhibitory molecule used. Contrasting results between the two studies also extend to the timing of repopulation. After oral administration of BLZ945, active microglia appeared 3 d posttreatment and normal numbers were reached at 7 d (Beckmann et al., 2018). Similarly, microglial depletion with PLX3397 in adult mice resulted in re-emergence of immature microglia 3 d after the cessation of treatment. These cells gradually acquired adult-like phenotypes and reached control density after 14 d (Elmore et al., 2014). In our study, microglia did not populate the MNTB until after 18 d posttreatment, at four weeks of age. The difference in repopulation timing likely lies in our method in elimination of microglia early during postnatal development when microglial cells should be proliferating and populating the entire brain. BLZ945 treatment not only depleted microglia but, in part, prevented them from completing their initial brainstem colonization.

### Origins of repopulating microglia

Microglia have a low turnover rate and can survive throughout the lifespan of an animal (Fuger et al., 2017). They can self-regulate and renew several times throughout life in rodents (Askew et al., 2017; Tay et al., 2017). We successfully eliminated microglia early in development and observed that, after cessation of treatment, microglia repopulate the brainstem. The origins of the reappearing brainstem microglia are not known. Studies implicate that repopulation can be achieved in one of three ways – proliferation of remaining microglia, stimulation of microglial precursors or infiltration of peripheral monocytes (for review, see Han et al., 2019). The process of repopulation likely depends on the method of depletion. It is possible that irradiation leads to an influx of macrophages from the periphery because of the disruption of the blood-brain barrier (BBB; Simard and Rivest, 2004). On the other hand, pharmacological depletion with drug-infused rodent diet could induce the proliferation from resident microglial progenitor cells such as nestin positive cells (Elmore et al., 2014). Additionally, proliferation of surviving microglial cells could support the microglial replenishment in the CNS. Genetic ablation of microglia (80%) using the Cx3cr1^CreER^-based system showed that repopulating microglial cells arose exclusively from an internal CNS-resident cell pool (Bruttger et al., 2015; Askew et al., 2017; Jakel and Dimou, 2017; Huang et al., 2018). Earlier it was shown that repeated subcutaneous BLZ945 injections resulted in complete microglial elimination in the brainstem (Milinkeviciute et al., 2019), thus, new cells do not likely emerge from surviving microglia. Peripheral macrophages could be another origin if the BBB was disrupted; however, despite the evidence that BLZ945 crosses the BBB (Pyonteck et al., 2013; Beckmann et al., 2018), there are no reports about disruption of the BBB. Consequently, CNS resident progenitors might be recruited to replenish the microglial cell pool after BLZ945 treatment is stopped. These studies support the view that the course of repopulation highly depends on the depletion approach used and the level of microglial elimination.

### Microglia and calyceal pruning

Microglia are continuously surveying their environment by extending and retracting their processes (Nimmerjahn et al., 2005). During development, microglia have been shown to have a significant role in the formation of neural circuits (Miyamoto et al., 2016; Reemst et al., 2016; Nelson and Lenz, 2017; Basilico et al., 2019; Nonaka and Nakanishi, 2019) in health and pathologic conditions (Neniskyte and Gross, 2017; Wolf et al., 2017; Whitelaw, 2018). Microglia can eliminate synapses (Paolicelli et al., 2011; Schafer et al., 2012) or promote their formation (Schafer et al., 2012; Parkhurst et al., 2013; Miyamoto et al., 2016). In MNTB, microglia appear at the time coinciding with intense synaptic development. They peak in number at around P14 and a slight decline is observed at P23 (Dinh et al., 2014). We noticed a further significant reduction in microglial areal coverage between weeks 4 and 7. This change may be reflective of a shift in the function of microglia from a developmental role to a homeostatic role.

In the auditory brainstem, microglial elimination resulted in increased numbers of polyinnervated neurons remaining in the MNTB after hearing onset (Milinkeviciute et al., 2019). It was not clear, however, whether the calyceal pruning was delayed or directly related to the absence of microglia from MNTB. Here, we found that even when treatment is stopped at P10 and microglia are allowed to populate the brain, innervation of principal MNTB neurons remains abnormal until microglia inhabit the nucleus. It has been shown that microglia promote brain repair after injury (Willis et al., 2020). In our case there was no injury, and microglia appeared in the MNTB between weeks 3 and 4 for the first time. It appears that newly emerged microglia initialized the developmental process that normally occurs before the onset of hearing. Depletion and subsequent recolonization of microglia in the retina showed restoration of functions of endogenous microglia in homeostasis (Zhang et al., 2018). It is important to note that calyces of Held developed normally in terms of their surface area and complexity; however, the pruning of additional calyces was impaired, indicating that there may be different mechanisms that regulate these two processes. These findings suggest a correlation between microglial presence and pruning of calyces of Held in MNTB.

### Microglia-astrocyte communication

Crosstalk between glial cells is essential for brain development and function in physiological and pathologic conditions. Bidirectional communication between microglia and astrocytes is established through cytokines, chemokines, ATP and growth factors early in development when they first populate the brain parenchyma (Jha et al., 2016; Liddelow et al., 2017; Vainchtein et al., 2018). Previously, it was shown that GFAP expression is reduced after microglia depletion (Milinkeviciute et al., 2019). Here, we found restoration of GFAP expression to control levels only after prolonged microglial repopulation. In contrast, oral BLZ945 treatment in the five-week cuprizone mouse model increased the number of GFAP**-**positive and ALDH1L1-positive astrocytes in the cortex, striatum, and corpus callosum (Beckmann et al., 2018). Similarly, PLX3397 treatment in adult mice resulted in a significant increase in GFAP mRNA (Elmore et al., 2014; Jin et al., 2017). This discrepancy may be attributed to the depletion method used; the delivery method; the developmental stage of the animal at the time of treatment; and/or the area of the brain analyzed. Here, microglia were depleted at the time when the brainstem should be undergoing the initial colonization by microglia (Dinh et al., 2014). This process was halted by injection of CSF1R inhibitor before microglia had a chance to occupy their positions and mature within the auditory brainstem. Given that GFAP is suggested to be a marker of mature (Wofchuk and Rodnight, 1995; Gomes et al., 1999; Middeldorp and Hol, 2011) astrocytes, it is possible that astrocytic maturation is impeded by microglial absence and when microglia are permitted to repopulate, GFAP expression is initiated and becomes comparable to that of control at seven weeks. It is yet to be determined which molecules may be involved in the process of astrocytic maturation.

### Auditory brainstem function

Auditory function requires precision in the development of neuronal circuits as well as accurate organization of specialized synapses. Microglia are known to be necessary for the formation of some neural circuits (Miyamoto et al., 2016; Basilico et al., 2019; Nonaka and Nakanishi, 2019). We thus examined the overall effect of microglia depletion on the auditory function. We found that temporary depletion of microglia in the brainstem early in development resulted in deficits in the ABR. BLZ945 treatment during the first postnatal week caused a moderate hearing loss but thresholds recovered by seven weeks, while click thresholds did not show recovery at this later time period. We also found that BLZ945 treatment had a significant effect on absolute peak latencies as well as interpeak latencies at most frequencies tested, and at seven weeks, these latency defects were still mostly present. However, the diminished amplitudes observed at four weeks in BLZ945-treated mice showed almost full recovery by seven weeks.

Our observation of elevated ABR thresholds could indicate a peripheral defect in treated mice. Defective CSF1R signaling affects bone resorption and leads to abnormalities in the middle and inner ears (Aharinejad et al., 1999; Kanzaki et al., 2011; Okano and Kishimoto, 2019). In the cochlea, CSF1R-positive macrophages are found in the spiral ligament, spiral ganglion and stria vascularis (Okano et al., 2008; Okano and Kishimoto, 2019) while centrally, microglia rely on CSF1 signaling for survival (Ginhoux et al., 2010; Erblich et al., 2011). Thus, treatment with CSF1R inhibitor can affect many areas in the auditory pathway. Our ABR results are consistent with hearing deficits reported in *Csf1^op/op^* mice where auditory thresholds were significantly elevated likely because of bone deformities and subsequent conductive hearing loss (Aharinejad et al., 1999; Okano and Kishimoto, 2019). We observed delayed absolute latencies in subsequent peaks as well as interpeak latencies, consistent with brainstem auditory evoked potentials recorded in *Csf1^op/op^* mice (Michaelson et al., 1996). Central defects could result in part from impaired peripheral function.

In addition to potential peripheral effects, our ABR analyses suggest that BLZ945 may produce central effects independently through its elimination of microglia. We observed significant increases in interpeak latencies in treated animals. A minimal effect was seen for peak I–II, but greater effects were seen for all of the central intervals. These delays were partially corrected after microglia repopulation. Peak III corresponds to the superior olivary complex and reflects in part activity in MNTB. However, it is not known to what extent calyceal pruning directly influences the latency or amplitudes of this peak in our study. Nevertheless, the observation of increased interpeak latency, impaired pruning, and delayed astrocyte maturation in MNTB together support a central effect of microglia elimination on auditory function.

Microglia have been shown to communicate with other glial cells, including oligodendrocytes (Lloyd et al., 2017) and NG2 cells (Müller et al., 2009; Giera et al., 2018) and regulate oligodendrocyte differentiation (Miron and Franklin, 2014). Microglial depletion has revealed a microglial role in the development and maintenance of oligodendrocytes and their progenitors (Hagemeyer et al., 2017). Oligodendrocytes are responsible for myelination of axons and, thus regulate the precise signal conduction seen in auditory pathways (Seidl, 2014; Sinclair et al., 2017; Long et al., 2018). In the MNTB, myelination starts by P9 (Saliu et al., 2014) and increases around the time of ear canal opening (Sinclair et al., 2017). Taken together, microglial elimination may affect conduction velocity (Sinclair et al., 2017).

We showed that treatment with CSF1R inhibitor temporarily reduced peak amplitudes, especially the central peaks. Thus, there are both peripheral and central defects in the ABR. On one hand, bone deformities in the middle and inner ear (Aharinejad et al., 1999; Kanzaki et al., 2011; Okano and Kishimoto, 2019) could have contributed to these impairments which then propagated to the central auditory system, however, we did not test this hypothesis directly. On the other hand, centrally, oligodendrocytes are known to detect and respond to neuronal activity (Gibson et al., 2014) as well as secrete neurotrophic factors. In the auditory brainstem, lack of BDNF secreted by oligodendrocytes significantly reduced amplitudes of ABR peaks II–IV in response to click stimuli (Jang et al., 2019). NG2 cells near calyces of Held were suggested to participate in regulating the fast-signaling properties of calyceal inputs (Müller et al., 2009). Thus, microglial elimination could have affected oligodendrocytes or NG2 cells, leading to impaired neuronal activity and synaptic synchrony which resulted in the reduction of amplitudes we observed.

The observed decrease in ABR amplitudes may be also related to delayed maturation of GFAP positive astrocytes. Astrocytes are highly fenestrated and interspersed between the branches of calyces (Ford et al., 2009; Holcomb et al., 2013). GFAP astrocytes express glutamate transporters and regulate glutamate uptake activity (Bergles and Jahr, 1997; Matthias et al., 2003), and are crucial for its recycling (Pow and Robinson, 1994; Laake et al., 1995; Hertz et al., 1999). These glial cells respond to glutamate release from the calyx of Held, which leads to the uptake of excess glutamate and prevention of glutamate receptor saturation, especially in the immature calyx (Bergles and Jahr, 1997; Matthias et al., 2003). In this paper, we showed that temporary microglia depletion during postnatal development reduced GFAP+ astrocyte levels. It is thus possible that delayed astrocytic maturation led to impairment in glutamate uptake and allowed for glutamate receptor saturation, and subsequent decrease in ABR amplitudes of the central peaks.

At this point, we cannot predict what exactly causes deterioration of different aspects and parts of ABRs as microglia are found through the auditory system. It is possible that macrophages in the cochlea and microglia in different auditory nuclei have slightly different functions. For peak I, which reflects peripheral activity, frequency-specific effects could reflect tonotopic differences in the cochlea of molecules that interact with macrophages. Centrally, there may also be differences in the way tonotopic regions of individual nuclei interact with glial cells.

Because of the fact that ABR peak III encompasses the auditory response of the entire SOC, we cannot correlate anatomic abnormalities observed in MNTB with ABR results. Rather, we can conclude that temporary loss of CSF1R signaling affects conduction velocity and strength throughout the ascending auditory pathway.

In conclusion, microglia function in a myriad of processes ranging from immune responses to synaptogenesis in the CNS. We eliminated this major cell population and showed remarkable plasticity in the developing auditory brainstem. Not only do microglia completely recolonize the brainstem, but repopulation rectifies previously observed anatomic defects. We showed that microglial presence in MNTB is needed in order for calyceal pruning to take place and for full establishment of the GFAP-positive astrocytic population. Importantly, microglial absence postnatally has a significant impact on auditory function, which partially recovers after microglia return.

## Synthesis

Reviewing Editor: Christine Portfors, Washington State University Decisions are customarily a result of the Reviewing Editor and the peer reviewers coming together and discussing their recommendations until a consensus is reached. When revisions are invited, a fact-based synthesis statement explaining their decision and outlining what is needed to prepare a revision will be listed below. The following reviewer(s) agreed to reveal their identity: Adrian Rodriguez-Contreras, Ivan Milenkovic.

The reviewers agree that the question of microglial contribution to the development of the auditory circuit in the brainstem is interesting and the novel result is potentially interesting to a broad audience. However, both reviewers agree that several points need to be clarified before the findings can be fully evaluated.

Reviewer 1:

In the present study the Milinkeviciute and colleagues used a CSF1R inhibitor to eliminate microglia with multiple injections in mice between P1 and P10. After the last injection at P10, the drug was withdrawn to track microglia repopulation and its effects on synaptic development in the brainstem medial nucleus of the trapezoid body (MNTB). Effects on auditory function were measured by auditory brainstem response electrophysiology. The authors report that absence of microglia does not affect the development of mono innervation and affects the expression of GFAP in astrocytes in the MNTB. Absence of microglia also affects the auditory brainstem response (ABR). After microglia repopulate the brainstem the GFAP and auditory defects are reversed.

Although the study is very interesting in scope, and the proposal that microglia "retain their developmental functions beyond the period of circuit refinement" is potentially a novel result that will be of interest to a broad audience, this reviewer found several points that need to be clarified before the findings can be evaluated.

1. Abstract, page 1, lines 11 and 12. The authors state that microglia recovered at 18 days after drug treatment. All data is reported at 3, 4 and 7 weeks. Please clarify.

2. Abstract, page 1, lines 13-15: I would suggest to include more specific information of what latencies and amplitudes were affected. Please include information that is relevant to understand how ABR results relate to effects in the MNTB.

3. Introduction; page 3, lines 73 to 74 state that microglia recolonized MNTB in lateral regions first, then in medial regions. What is the evidence to support such a claim?, and if this can be confirmed by analyses of existing data, how does it relate to the effects observed on ABRs?

4. Materials and methods. Animals and treatment.

a) Please clarify how many animals were used at P18.

b) Clarify what is the control condition.

c) Clarify what is the concentration of BLZ945 stock solution used for injections.

d) Clarify if mice were sacrificed when aged 3, 4 and 7 weeks; or if mice were sacrificed 3, 4 and 7 weeks after P10.

5. Auditory brainstem recordings.

a) The authors point that the MNTB is involved in sound localization. Why did the authors decide on ABR versus interaural intensity difference (ILD) measurements? A more clear justification of the use of ABRs is needed.

b) It is not clear what was the duration of pure tone stimuli.

c) It is not clear what was the duration of ABR recordings. The authors state 3 ms (page 7, line 180), but report longer latencies in Figures 5 and 6.

6. ABR analysis.

a) I would suggest a more objective definition of ABR threshold. A definition that considers signal to noise ratio may be useful. See: Bogaerts et al. BMC Neurosci. 2009; 10:104; or for a different approach see Suthakar and Liberman. Hear Res. 2019; 381:107782.

b) What is the relevance of the interpeak latency in this study?

c) The slope of I/O functions should be determined and reported in the corresponding figure. See Sinclair et al 2017 (figure 9) who analyzed wave IV latency for MNTB. Can this help the authors interpret the functional deficits against the anatomical findings?

7. Results.

a) Page 9, lines 211 and 212. These two sentences should be in the methods.

b) An inconsistency was noted. Page 9 lines 212 and 213. Iba1 immunofluorescence was not observed at 3 weeks after last drug injection. The abstract states microglia returned at P18. If Figure 1 is correct, then microglia repopulate the brainstem between weeks 3 and 4.

c) Fig 1. What is the significance of decrease in microglia in controls between 4 weeks and 7 weeks?

d) Fig 2. Was there a difference in GFAP between week 4 and week 7 in controls?

e) Results in Figures 1 and 2 suggest a relationship between Iba1 and GFAP fluorescence. However this point is not addressed by experiment. Authors could test this idea with a double staining and a plot of Iba1 vs GFAP intensity or areal coverage ratios. This point is also related to the statement that microglia come back in lateral regions before the medial regions of MNTB (see point 3 above).

f) In fig 3 no difference in % polyinnervated neurons was observed at week 4. This suggests that pruning occurs before microglia repopulation takes place. What is the mechanism of pruning without microglia? In my opinion the 3 week time point is needed to interpret data at 4 weeks. It may be useful to test if microglia engulf calyx of Held synapses at week 3 compared to week 4.

g) Figures 4, 5 and 6. In general, it would be useful to include waveforms to illustrate the main points relevant to understand the relationship between microglia ablation and auditory defects. For example: Figure 5. What is the interpretation of a longer latency of wave 1 at 8 kHz and a shorter latency of wave 1 at 32 kHz for treated mice versus controls, week 4 data?

h) It is not clear why controls at 4 weeks have such low click thresholds if 10 dB was the softest click played to animals. What about pure tone data where thresholds are at 10 dB for 4 kHz, and above 10 dB for 8-32 kHz frequencies. It would be useful to show ABR traces indicating the differences in thresholds between conditions.

i) The distribution of ABR click thresholds is generally normally distributed. This is not the case for control data at 4 wk and for treatment data at 7 wk. This is particularly interesting for the treated 7 week data since some mice seem to recover while most have elevated thresholds. What is the relationship between click thresholds and pure tone thresholds? This data is available since the same mice were tested with both stimuli. This is important since medial versus lateral MNTB regions respond to different sound frequencies.

j) It is not clear if mice used for ABR were also used for histology. See my previous comment about some animals having low versus some animals having high thresholds within the treated condition. Is there a correlation between the histology and the functional defects?

8. Discussion.

a) The statement on page 14 Lines 335 to 337 may need to b revised (see comment 7f above).

b) Page 17 Line 432. You mean "affect conduction velocity."

c) The discussion is thorough review of literature, but I did not understand what is the main conclusion based on the data: is drug treatment causing a systemic effect on microglia and other cellular populations in the periphery? or is the effect localized in the CNS? What is the link between ABRs and MNTB pruning/astrocyte defects?

Reviewer 2:

The major findings can be summarized as follows:

- The MNTB completely lacks microglia at 3wk, while it recovers quickly by 4wk and remains at control level at 7wk

- GFAP labeling as a marker of astrocytes is lagging the recovery of microglia reaching the control level at 7 wk

- The recovery of microglia correlates with the reduction of % of polyinnervated neurons (compared to ∼40% at P13 from Milinkeviciute et al., 2019). Interestingly, the incidence of polyinnervated neurons at 4wk is not significantly different from control, yet the ABR recordings indicate deficits in auditory processing.

-ABR measurements show increased thresholds to clicks both at 4wk and 7wk, and also to pure tones for 4-24kHz at 4wk and for 4 and 8kHz at 7wk.

-Other ABR parameters, such as peak latency, peak to peak latency, and wave I-IV amplitudes show some changes, gradually decreasing from 4 to 7wk and from 8kHz to 32kHz for a given age.

The experiments have been well conducted, the presentation of data is good, and statistical analyses are appropriate and solid. The tables showing full statistical analyses are well appreciated.

While the main message of the study is generally backed by the ABR data, it if difficult to draw more detailed conclusions regarding the potential deficits in the signal processing based on the present results. One wishes a more sensitive functional analysis in addition, or instead, particularly assessing the calyx of Held-PN synapse.

Nevertheless, the interesting data potentially offer a novel insight into the microglial contribution to development of the auditory circuit in the brainstem, providing a solid base for follow up investigations.

There are few points requiring authors’ attention:

Major

1) It is interesting that microglia fully repopulate the MNTB between P21 and P28. Also, the percentage of polyinnervated PNs is similar between BLZ and control at 4wk. Finally, the calyx surface area is similar between the two groups (Fig. 4G), possibly indicating similar fenestration. From this, one may postulate the recovery of function at the calyx synapse. However, the ABR recordings at 4wk show an increased latency of peak III for low, mid, and high frequency, and also a reduced peak III amplitude for low and mid frequencies. Some of these parameters remain different from control even at 7wk. The recovery of auditory function as stated at several places in the manuscript does not temporally match the suggested morphological rectification. It remains unclear to what extent the rather solid morphological data at the calyx of Held synapse can explain the observed ABR changes. This question deserves to be addressed in the discussion, in addition to several other mechanisms suggested, not directly referring to synaptic function.

2) The effects of BLZ treatment were more prominent in the low to mid frequency range. Can authors discuss why these projections may be more sensitive to microglia depletion?

Minor:

1) The reference Brand et al 2002 (line 38) does not seem appropriate for the integration of excitation and inhibition by the LSO neurons in the IID circuit. Brand et al investigated the effect of inhibition on the MSO neurons in the ITD circuit. The same applies for the following sentence in line 39. The results in Fig. 2E-G are only mentioned in the text after description of the data for Figure 3. It would be more intuitive to move these data to Figure 3.

2) Line 85: please include Author University

3) Line 408: the sentence does not faithfully reflect the data: "only high frequency thresholds recovered by 7wk".

Threshold at 32kHz is not affected at all at 4 weeks, and thresholds in the mid frequency range 12 and 16 kHz recover by the 7th week. At 7 weeks, only the thresholds at 4 and 8 kHz remain elevated. Please correct.

4) Line 435: The sentence states peripheral and central defects as a consequence of CSF1R inhibition. While peripheral changes may be postulated according to ABR recordings, there is no solid data in this study to support such conclusion. The authors may want to tune down this statement.
